# Digital Cognitive Behavioral Therapy–Based Treatment for Insomnia, Nightmares, and Posttraumatic Stress Disorder Symptoms in Survivors of Wildfires: Pilot Randomized Feasibility Trial

**DOI:** 10.2196/65228

**Published:** 2025-03-14

**Authors:** Fadia Isaac, Britt Klein, Huy Nguyen, Shaun Watson, Gerard A Kennedy

**Affiliations:** 1 Institute of Health and Wellbeing Federation University Australia Victoria Australia; 2 Health Innovation & Transformation Centre Federation University Australia Ballarat Australia; 3 Department of Population and Quantitative Health Sciences UMass Chan Medical School Worcester, MI United States; 4 Department of Epidemiology Institute for Preventive Medicine and Public Health Hanoi Medical University Hanoi Vietnam; 5 Faculty of Health Sciences University of Cuu Long Vinh Long Province Vietnam; 6 School of Health and Biomedical Sciences Royal Melbourne Institute of Technology University Melbourne Australia

**Keywords:** insomnia, nightmares, posttraumatic stress disorder, PTSD, wildfires, cognitive behavioral therapy for insomnia, CBTi, exposure, relaxation, and rescripting therapy, ERRT, Sleep Best-i, mobile health, mHealth, digital health, computer, eHealth, bushfires

## Abstract

**Background:**

Symptoms of insomnia, nightmares, and trauma are highly prevalent. However, there are significant barriers to accessing evidence-based treatments for these conditions, leading to poor mental health outcomes.

**Objective:**

This pilot trial evaluated the feasibility of a 4-week, digital self-paced intervention combining cognitive behavioral therapy for insomnia and exposure, relaxation, and rescripting therapy for nightmares in survivors of wildfires from Australia, Canada, and the United States.

**Methods:**

Study participants were recruited between May 2023 and December 2023 through social media platforms, workshops, conferences, and radio interviews. Participants had to meet at least one of the following criteria: a score of ≥8 on the Insomnia Severity Index, a score of ≥3 on the Nightmare Disorder Index, or a score of ≥31 on the PTSD Checklist for DSM-5. In total, 30 survivors of wildfires were allocated to either the treatment group (n=16, 53%) or the waitlist control group (n=14, 47%) in a sequential manner. Participants’ ages ranged from 18 to 79 years, with a mean age of 52.50 (SD 16.26) years. The cohort consisted of 63% (19/30) female and 37% (11/30) male participants. Participants also completed self-report secondary outcome measures, including the Generalized Anxiety Disorder–7, the Patient Health Questionnaire–9, and the Pittsburgh Sleep Quality Index, via the HealthZone digital platform. Assessments were conducted at baseline, the posttreatment time point, and the 3-month follow-up, with the waitlist group undergoing an additional assessment at the pretreatment time point, after 4 weeks of waiting and before crossing over to treatment. This study used intention-to-treat analysis as a primary analysis and per-protocol analysis as a secondary analysis.

**Results:**

Mixed-effects linear regression models and difference-in-differences analyses were used to assess the intervention’s effects. The intention-to-treat analysis revealed significant improvements over time (main effect of time), with a 1.64-point reduction (*P*=.001) on the Nightmare Disorder Index and 10.64-point reduction (*P*=.009) on the PTSD Checklist for DSM-5 at the postintervention time point. No significant changes were observed in insomnia symptoms. On the secondary measures, there was an interaction effect of condition × time, with a 2.22-point reduction (*P*<.001) on the Pittsburgh Sleep Quality Index, and a main effect of time, with a 6.48-point reduction (*P*<.001) on the Patient Health Questionnaire–9. No changes were detected on the Generalized Anxiety Disorder–7. The per-protocol analysis yielded comparable results for both the primary and secondary measures.

**Conclusions:**

The findings of this pilot trial demonstrated a reduction in nightmares and trauma symptoms. Future research studies should aim at evaluating the intervention in a more definitive trial with a larger sample size.

**Trial Registration:**

Australian New Zealand Clinical Trials Registry (ANZCTR) ACTRN12623000415606; https://anzctr.org.au/Trial/Registration/TrialReview.aspx?id=385054

## Introduction

### Background

The consequences of wildfires can be devastating, resulting in significant losses of life, property, and livelihood. According to the Emergency Events Database, wildfires have been responsible for 1890 fatalities and 14,360 injuries between 2000 and 2023, with the highest tolls observed in Australia, New Zealand, Southern Europe, and North America [1]. The impact of wildfires extends far beyond the immediate loss of life and property, with survivors often experiencing a range of physical and emotional challenges that can have long-lasting effects. Physical injuries, such as bodily burns, smoke inhalation, and eye irritation, as well as increased risk of myocardial infarction and certain types of cancers, are a significant concern for survivors [2-4]. In addition, the proximity of wildfires to residential areas can have a profound impact on overall well-being, with research showing that life satisfaction decreases significantly in areas within 0 to 15 km of the fires [5]. The traumatic experience of a wildfire can also leave a lasting emotional impact, with survivors often reporting persistent feelings of fear and unsafety that can linger long after the initial event [5].

Beyond the physical and emotional challenges, many survivors face significant financial pressures, including the cost of rebuilding or relocating. For those who have lost their homes, the decision of whether to rebuild can be a difficult and emotional one, with financial pressures and the loss of a sense of security and community adding to the burden [3]. Others may experience stressors related to the loss of a workplace, hardship, and financial uncertainty. Therefore, the availability and timing of government relief and community support services are critical in facilitating the recovery of communities and individuals, with initiatives such as rehousing and recovery projects, psychological support, and primary producer repair and restoration [3,5] helping mitigate the impact of wildfires. However, when such support is not provided in a timely manner, emotional and psychological distress can intensify, leading to multiple psychological problems that can become highly resistant to treatment.

This is particularly concerning as research has shown that exposure to wildfires can have a significant impact on emotional well-being. Over the past decade, there has been a growing interest in investigating the impact of wildfire trauma on mental health [6,7]. Posttraumatic stress disorder (PTSD) is among the more severe mental health conditions that may arise immediately or in the months following exposure to traumatic events [7]. Exposure to wildfires, which pose a significant threat to life or well-being, can increase an individual’s risk of developing PTSD. People with PTSD may exhibit symptoms such as reliving traumatic events through nightmares or flashbacks; sleeping difficulties; irritability; intrusive memories; feelings of horror, shame, and anger; avoidance of reminders of the traumatic event; alterations in arousal state or mood and cognition; and difficulties with work and daily activities [8].

Sleep disturbances are prominent features of PTSD and are frequently reported following trauma [9,10]. The onset of sleep disturbances following exposure to traumatic events is a strong predictor of the development of PTSD [11]. According to the *Diagnostic and Statistical Manual of Mental Disorders, Fifth Edition* (*DSM-5*), insomnia and nightmares fall under the re-experiencing and the hyperarousal clusters of PTSD symptoms [8]. Insomnia is the most prevalent sleep disturbance in survivors of wildfires and is characterized by difficulty initiating or maintaining sleep or early-morning awakening [8]. Survivors of trauma also frequently experience nightmares, which are repeated distressing and well-remembered narrative dreams that lead to the individual waking up in a fight-flight state of alertness and having difficulty returning to sleep or even trying to avoid sleeping [8]. A study examining sleep disturbances and PTSD in survivors of wildfires revealed that approximately 49.2% (n=126) experienced symptoms of insomnia, 28.7% experienced nightmares, and 77.9% reported PTSD symptoms [12].

Studies suggest that cognitive behavioral therapy for insomnia (CBTi); cognitive behavioral therapy (CBT) for PTSD; and exposure, relaxation, and rescripting therapy (ERRT) for nightmares are effective treatments for insomnia, nightmares, and other trauma symptoms [13-18]. CBTi includes stimulus control, sleep hygiene, sleep restriction, cognitive restructuring, and relaxation training [19,20]. Nightmare treatment using ERRT is effective in giving those who experience nightmares a sense of control over the nightmare through writing benign dreams that can be rehearsed verbally or mentally [21]. The treatment focuses on addressing the themes presented in the nightmare and incorporates rescripting, imagery or verbal rehearsal, relaxation, and sleep hygiene elements [22,23].

Most research on trauma and sleep disorders has been conducted with military veterans, who have been the primary focus of clinical trials and studies in this area [24-26]. There is a need to focus on developing treatments for people who have experienced the trauma of natural disasters, including wildfires. There have only been 2 clinical trials addressing the treatment of sleep disturbances and PTSD in survivors of wildfires [27,28]. Both trials have shown that the administration of sleep dynamic therapy and CBTi therapist-assisted self-help interventions led to a significant reduction in insomnia and PTSD symptoms following treatment.

### Objectives

While studies support the effectiveness of CBT, CBTi, and ERRT in treating trauma symptoms, insomnia, and nightmares, accessing these treatments can be challenging, particularly when wildfires cause destruction to infrastructure. This, in turn, causes difficulty in the delivery of counseling services to remote locations where large numbers of people are affected and in great need. Digital therapies can bridge this gap by delivering evidence-based treatments in communities affected by the trauma of wildfires [29,30]. However, the feasibility of digital therapies should be first examined in clinical trials before they are operationalized in the field [27,31]. Therefore, a brief, digital, self-paced, multicomponent therapeutic approach incorporating CBTi for insomnia, CBT for PTSD, and ERRT for nightmares was developed. The main objective of this pilot trial was to assess the feasibility of an intervention called Sleep Best-i in alleviating symptoms of insomnia, nightmares, and PTSD among survivors of wildfires. A secondary research objective was to undertake a comparative analysis of the degree of symptom reduction between the intervention and the waitlist control groups, with a view to informing an assessment of the feasibility of conducting a future definitive randomized controlled trial. Specifically, this assessment aimed to evaluate the acceptability and usability of the Sleep Best-i intervention. This clinical pilot trial tested the following hypotheses: (1) Sleep Best-i will result in significant reductions in insomnia, nightmares, and PTSD symptoms from baseline to the posttreatment time point compared to the waitlist group; (2) Sleep Best-i will also lead to significant reductions in anxiety and depression scores and improvements in sleep quality from baseline to the posttreatment time point compared to the waitlist group; and (3) both groups (within-group effects) will experience significant improvements in all measures (insomnia, nightmares, PTSD symptoms, anxiety, depression, and sleep quality) from baseline to the posttreatment time point, and these improvements will be maintained at the 3-month follow-up after receiving Sleep Best-i.

## Methods

### Study Design

This study used a parallel-arm, sequential alternation method of randomizing participants to either the intervention or waitlist group in a 50:50 ratio, with a crossover of the latter group. The alternation method ensures the ability to answer questions about the effectiveness of treatments to inform decision-making in clinical practice [32]. The treatment group completed self-report assessments at baseline, at the posttreatment time point following the administration of Sleep Best-i, and at the 3-month follow-up. The waitlist group completed the same assessments at baseline, at the pretreatment time point (following 4 weeks of waiting and before crossing over to treatment), at the posttreatment time point following the administration of Sleep Best-i, and at the 3-month follow-up. The CONSORT (Consolidated Standards of Reporting Trials) checklist was followed [33]. Please refer to Appendix 1 for the CONSORT-EHEALTH (Consolidated Standards of Reporting Trials of Electronic and Mobile Health Applications and Online Telehealth) checklist (Multimedia Appendix 1).

### Ethical Considerations

Following ethics approval from the Federation University Human Research Ethics Committee (approval 2022-153), an advertisement about the study was distributed. Survivors of wildfires interested in the study read a plain-language information statement (PLIS) about the study and provided baseline data. Participants who met the selection criteria provided informed consent by checking a box on a digital consent form hosted on Federation University’s HealthZone platform, thereby indicating their agreement to take part in the study. Participants were informed that their participation was voluntary. To ensure the secure storage and handling of collected information, data were stored on a password-protected digital health platform (HealthZone) during the trial accessible only to the researchers involved in the study. To protect participants’ confidentiality, names were replaced with unique identifiers, and only country of residence was collected, with no addresses recorded. The data will be retained for 15 years before being destroyed. To manage any potential risks, participants were provided with emergency contact numbers relevant to their country of residence. As a gesture of appreciation, participants who completed the study were offered an Aus $100 e-voucher (approximately US $66.70).

### Participants

Participants were recruited between May 2023 and December 2023 via social media platforms, including international Facebook campaigns, radio broadcasts, LinkedIn, Reddit, and online community noticeboards. This study was also advertised on the Natural Hazards Research Australia and the Australian Institute for Disaster Resilience websites.

Adult survivors of wildfires from Australia, Canada, and the United States aged ≥18 years were invited to take part in the clinical pilot trial if they had experienced wildfires at any point in the past and had sleep or trauma symptoms.

An a priori power analysis indicated that a sample size of 50 was required to detect meaningful differences between the intervention and waitlist groups considering an α value of .05, a value of *d* of 1.0, an attrition rate of 20%, and 80% power [34].

### Inclusion Criteria

To be eligible to take part in the trial, participants had to (1) provide digital consent; (2) have a score of ≥8 on the Insomnia Severity Index (ISI), (3) a score of ≥3 on the Nightmare Disorder Index (NDI), or (4) a score of ≥31 on the PTSD Checklist for DSM-5 (PCL-5); (5) be fluent in the English language; and (6) have access to the internet. Exclusion criteria included not being a survivor of a wildfire, diagnosis of a psychotic disorder, suicide risk, diagnosis of sleep apnea or restless leg syndrome, diagnosis of alcohol or drug dependence, and attendance to psychotherapy for either sleep or PTSD conditions. In addition, participants were excluded if they were currently using steroids for any health condition; medications that promote sleep, such as benzodiazepines (eg, alprazolam, clonazepam, and temazepam); or medications that may affect sleep, such as opiates or other pain medications. By excluding these individuals, this study aimed to ensure that participants were suitable for the trial and that the results would not be confounded by other factors.

### Procedure

The trial was registered prospectively in the Australian New Zealand Clinical Trials Registry (ACTRN12623000415606). Since its initiation, this study’s protocol has undergone only minor revisions, specifically an update to the statistical analysis methodology to include linear mixed methods and intention-to-treat (ITT) analysis.

An advertisement of this pilot study with a URL was distributed on social media platforms displaying Federation University and the Natural Hazards Research Australia affiliations. This study was also promoted in national conferences, sleep workshops delivered to communities affected by wildfires, the Red Cross, and group meetings for people with sleep disorders in Australia. Radio interviews were also undertaken to promote the study. Survivors of wildfires who were interested read a PLIS (refer to Multimedia Appendix 2 for PLIS); signed a digital consent form (refer to Multimedia Appendix 3 for the consent form) to register an account on Federation University’s digital HealthZone platform; and provided baseline data by completing a demographic questionnaire as well as self-report measures on insomnia, nightmares, and trauma symptoms. Participants had the option of providing a pseudonym if they wished. However, they needed to provide a valid email address to allow for communication about the study. Their email addresses were also directly connected to their personal dashboard on HealthZone. The baseline data were viewed by 2 researchers to decide eligibility. Those who were eligible (assessed by FI and GAK, both clinical psychologists) were contacted via email to notify them of their eligibility and their randomization and receive instructions on how to access their personal dashboard on HealthZone. Participants were sequentially allocated via simple randomization in the order of their enrollment (using a computer-generated simple randomization sequence), and they were informed about the purpose, allocation, and structure of the study as explained in the PLIS. However, they were not informed about the study’s hypotheses. Once participants gained access to their personal dashboard within HealthZone, they were able to access information about the trial, complete the self-report secondary measures, and access the treatment modules consecutively. The treatment modules were provided at no cost, allowing participants to use their own devices and access the program and its modules on various platforms, including mobile devices, tablets, and desktop computers. HealthZone features a responsive web design compatible with both iOS and Android operating systems, making it easily accessible. Participant response data were captured on all devices. The modules were released sequentially to participants over 4 weeks. All data were collected through self-report measures at prespecified intervals. Participants were instructed to complete a module or a set of 2 modules each week along with the required interval assessments. Automated email reminders alerted participants about the release of each module and the specified assessments. If the modules were not viewed within the first 3 days of their release, an automated email reminder was sent to participants, and a second reminder was sent on the seventh day of their release. Automated email reminders at a similar frequency were also used for the primary and secondary measures (self-report scales). The personal dashboard was accessible to participants at any time. A “Contact us” tab was available on the personal dashboard that allowed participants to contact the research team and ask questions about the study or express any concerns. A total of 7% (2/30) of the participants were unsure about how to access the modules, and 3% (1/30) had an inquiry about one of the modules.

The research team sent 2 follow-up emails spaced 2 weeks apart to participants who registered, were randomized, but did not commence treatment, serving as a reminder about the study and to gauge their ongoing interest in participating.

The modules were available to participants from the start of the trial to the end of the follow-up period. Participants were able to access their dashboard for 4 weeks following the 3-month follow-up data collection. HealthZone recorded data about the number of log-ins, number of pages visited, and date of commencement and duration of participation for each participant. Each module was 17 minutes in duration. Therefore, participants were expected to spend at least 3 hours over the 4 weeks to indicate adherence to treatment. This time is optimal for viewing all modules and completing all assessments. No statistical analyses were conducted in the interim of the study. HealthZone was monitored daily during the trial to track any potential concerns or issues associated with the site or the participants.

### Treatment Protocol

#### Sleep Best-i Intervention

Sleep Best-i was specifically designed for this clinical trial by some of the researchers who conducted this study (FI, BK, and GAK). The collated treatment manual draws from evidence-based treatment manuals authored by other sleep researchers [19,21,35-39]. The main therapeutic methods used in the manual were CBTi and ERRT. The recorded digital modules feature human-recorded voice and animated videos using VideoScribe (Sparkol) [40] that explain concepts related to sleep and trauma symptoms. The intervention also offers 2 role-plays of therapeutic sessions to demonstrate the administration of cognitive restructuring, dream rescripting, and sleep scheduling. Sleep Best-i consists of 6 modules administered over a 4-week period. In addition, the program is accessible via a platform that uses a responsive web design, with no requirement for a specific app or download. This allows participants to access the program from a variety of devices (including mobile phones), as well as their preferred web browser. By being web based, the program can be easily accessed from anywhere at any time, making it a convenient and flexible intervention.

Participants were encouraged to complete the entire Sleep Best-i program, but completion of each module was not mandatory. This allowed individuals to engage with relevant content tailored to their specific needs, whether they experienced nightmares, PTSD, insomnia, or a combination of these conditions. Furthermore, participants had the flexibility to stop and start the program as desired, and all modules were available for the entire study period, enabling them to revisit specific modules should they wish to do so.

Table 1 shows the therapeutic modality offered in each module. Once released, the modules remained unchanged throughout the trial, and the intervention was successfully implemented without encountering technical difficulties. There were periodical checks to ensure that the modules were compliant with the treatment manual. All links have been archived in the Wayback Machine (Internet Archive).

**Table 1:** Sleep Best-i modules and the treatment offered in each module. All modules were approximately 17 minutes in duration.

Module	Strategies offered
Module 1 (psychoeducation)	Offered at week 1, the module provided psychoeducation about sleep and insomnia, the stages of sleep, and the neurobiology of sleep [14].
Module 2—part 1 (cognitive restructuring and sleep hygiene)	Offered at week 1, the module focused on the cognitive component of CBTi^a^, types of unhelpful thoughts, methods to challenge unhelpful thoughts, and sleep hygiene [30].
Module 2—part 2 (sleep scheduling and stimulus control)	Offered at week 2, the module educated participants about specifying regular sleeping and waking up times with as little variation as possible between the 2. Stimulus control restricted the bedroom or the bed to sleep only [34].
Module 3 (trauma, PTSD^b^, and flashbacks)	Offered at week 3, the module provided psychoeducation about trauma, PTSD, flashbacks, and how trauma leads to sleep difficulties. It also provided behavioral interventions for trauma symptoms.
Module 4 (nightmares)	Offered at week 4, the module explored nightmare disorder, how nightmares develop, and how to rescript the nightmare into a more neutral dream [16].
Module 5 (relapse prevention)	Offered at week 4, the module focused on identifying early warning signs and methods preventing relapse of symptoms [33].
Mindfulness module	This module offered a recorded progressive muscle relaxation mindfulness, and it was available to participants throughout the study.

^a^CBTi: cognitive behavioral therapy for insomnia.

^b^PTSD: posttraumatic stress disorder.

#### Waitlist Control Group

Following a waiting period of 4 weeks, the waitlist control group received Sleep Best-i in the same sequence in which it was released to the treatment group.

### Measures

#### Overview

All scales were digital, self-report measures that were administered through HealthZone. The self-report measures as well as the modules were tested by 2 research team members (FI and BK) before being released to participants. The order of the modules and the questions were the same for all participants in the 2 groups.

#### Demographic Data

Participants provided the following information: name, email address, age, sex, employment status, educational level, marital status, country of residence, experience with wildfires, history of taking steroids, diagnosis of sleep apnea or restless leg syndrome, diagnosis of a psychotic disorder, use of alcohol or drug dependence, type of medications used to assist with sleeping, history of antidepressant use, and whether they were attending psychotherapy for sleep or PTSD.

#### Primary Measures

##### ISI Measure

The ISI [41] includes 7 items that assess insomnia severity through the subjective experience of sleep. The score varies from 0 to 28, with higher scores indicating more severe insomnia symptoms. A score of ≥8 indicates the presence of a subthreshold insomnia. Previous studies have demonstrated strong internal consistency for the ISI, with Cronbach α values ranging from 0.87 to 0.92, indicating high reliability [42].

##### NDI Measure

This scale consists of 5 items that assess symptoms of nightmares according to the DSM-5 criteria by screening for the occurrence of nightmares in a given week. The 5 items are summed to obtain a score between 0 and 20, with higher scores representing greater nightmare severity. The NDI exhibits good internal consistency, with a Cronbach α of 0.80 [43].

##### PCL-5 Measure

The PCL-5 [44] includes 20 self-report items assessing the presence of PTSD symptoms over a 1-month interval. A global score that ranges between 0 and 80 is obtained by summing the 20 items. A cutoff score of 31 provides optimal sensitivity and specificity in providing a provisional diagnosis of PTSD [45]. The PCL-5 has good psychometric properties, with an internal consistency of Cronbach α=0.94 [46].

#### Secondary Measures

##### Generalized Anxiety Disorder–7

This scale is a 7-item, self-report measure that assesses anxiety symptoms over a 2-week interval. A score from 0 to 21 is obtained by summing the 7 items. The Generalized Anxiety Disorder–7 (GAD-7) [47] has a good internal consistency, with a Cronbach α ranging between 0.82 and 0.93 [48]. A cutoff score of 10 discriminates between mild and severe symptoms of anxiety [48].

##### Patient Health Questionnaire–9

This scale consists of 9 items assessing symptoms of depression over the last 2 weeks. The overall score is obtained by summing all the 9 items and ranges from 0 to 27. A cutoff score of 10 is considered optimal in discriminating between mild and severe symptoms of depression [49]. In this study, the Cronbach α for the Patient Health Questionnaire–9 (PHQ-9) was 0.86.

##### Pittsburgh Sleep Quality Index

The Pittsburgh Sleep Quality Index (PSQI) [50] consists of 19 items assessing sleep quality and disturbances related to sleep over a 1-month interval. A global score ranging between 0 and 21 is generated by summing 7 components of sleep quality. A global PSQI score of >5 is considered a sensitive and specific cutoff to discriminate between good and poor sleep quality [51]. The PSQI has a good internal validity, with the Cronbach α ranging between 0.79 and 0.81 [51].

##### Satisfaction and Level of Engagement With Treatment

One question was designed for this study to measure satisfaction with Sleep Best-i. Participants were asked to rate how likely they were to revisit the Sleep Best-i modules on a 5-point Likert scale from *strongly disagree* to *strongly agree* (0-4). To assess level of engagement with treatment, the number of log-ins and amount of time that each participant spent on the site were monitored.

### Data Analysis

Data analyses were conducted using the Stata statistical software package (version 18; StataCorp) [52]. All variables were inspected for normality and outliers. An inspection of histograms and *Q*-*Q* plots revealed normally distributed data. Upon inspection of box plots on all scales, 1 outlier was identified on the PCL-5 at the 3-month follow-up assessment, a second outlier was detected on the PHQ-9 at the 3-month follow-up, and a third outlier was detected on the PSQI at the waitlist assessment (3-month follow-up assessment). The results did not differ upon removing the outliers from the analyses; therefore, the outliers were retained [53,54]. To ensure a robust evaluation of the intervention’s feasibility, the ITT analysis with 30 participants and the per protocol (PP) analysis with a sample of 20 were used, allowing for a comprehensive assessment of treatment outcomes, thereby enhancing the accuracy and generalizability of the findings [33,55,56].

For both analyses, missing data were addressed using multiple imputation by chained equations. This approach is more robust than other methods such as single imputation because it generates multiple predictions for each missing value, accounting for uncertainty in the imputations and yielding more accurate SEs [57]. To impute missing data, background variables such as age, sex, educational level, employment status, country, and relationship status; baseline measures; and the most recent observations of each outcome were used.

To address uncertainty, each missing value was imputed 20 times using predictive mean matching based on the 3 nearest values across multiple iterations to create 100 imputed datasets. The imputed values were then averaged to create the final estimates [58]. Predictive mean matching, a partially parametric method, is preferable to fully parametric linear regression as it remains effective even when the normality assumption of the underlying variable is violated. This method also helps preserve the distribution of observed values in the missing data [59]. Missing value analysis indicated that the data were missing completely at random (Little missing completely at random test: χ^2^_867_=0.0, *P*>.99), meaning that the differences between the missing and observed data were related to observed characteristics [60].

Adjusted analyses of intervention effects were conducted using mixed-effects linear regression models. Both fixed and random effects were estimated to assess the impact of the intervention on the change in all outcomes (primary and secondary) over time and by condition. Given that observed covariates were balanced between the 2 conditions (waitlist and treatment), the difference-in-differences (DID) effect for the fixed part of the mixed-effects models was tested by including interaction terms for time point and condition. DID is considered the most appropriate measure to assess causal effects in time-series designs as it effectively controls for time-invariant confounding variables. By doing so, DID reduces bias and enhances internal validity, providing a more accurate estimate of the treatment effect [61].

The appropriateness of the mixed-effects model for each outcome was assessed using chi-square statistics (*P*<.05). Random-intercept linear models were compared with random-intercept, random-slope models using likelihood ratio tests. The random-intercept, random-slope model was reported only if the likelihood ratio test indicated *P*<.05; otherwise, the simpler random-intercept model was selected. A significant effect of the intervention or difference in outcome between groups was assessed using a 2-tailed test of significance with *P*<.05.

A sensitivity analysis was conducted to test whether the model and covariance structures were misspecified. Several major types of covariance structures, including independent, unstructured, exchangeable, identity, and autoregressive structures, were examined. In addition, the model parameters using both maximum likelihood and restricted maximum likelihood methods were estimated. Although the results were generally consistent across different covariance structures, the model with an exchangeable structure was deemed the best fit.

### Clinical Significance

Clinical significance was assessed for the 2 groups from baseline to the 3-month follow-up. To evaluate the clinical significance on sleep and trauma measures, specific criteria were used for each scale. For example, for the ISI, a cutoff score of ≤8 with a ≥6-point reduction in scores was used to assess whether participants reached a minimal clinically significant change (MCSC) [26,62]. For the NDI, there are no established criteria for MCSC; therefore, a cutoff score of ≤7 as suggested by Dietch et al [43] was used. Marx et al [63] recommend a score of ≤28 and a drop of ≥18 points on the PCL-5 as a guide for MCSC.

## Results

### Overview

**Figure 1:**
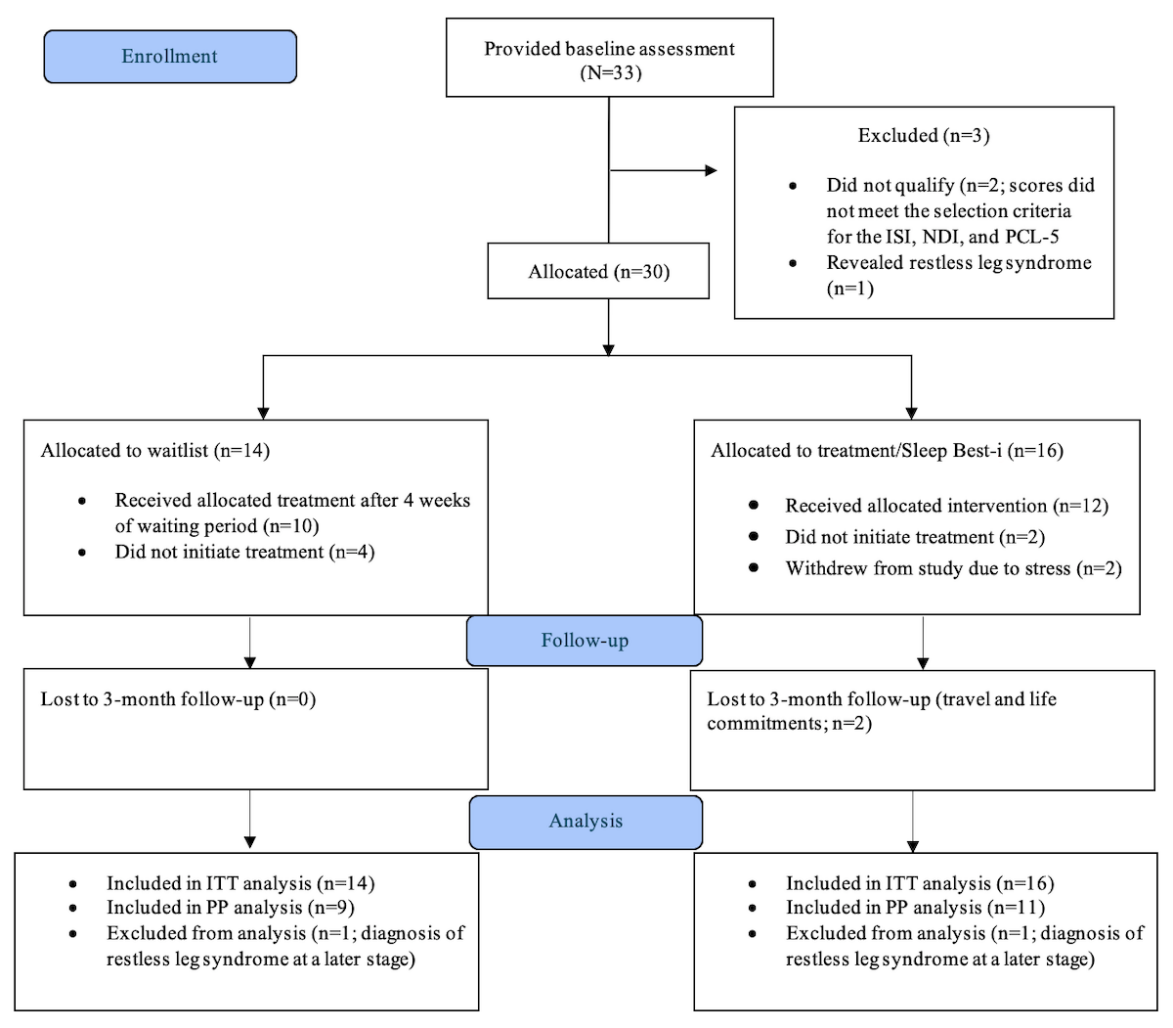
CONSORT (Consolidated Standards of Reporting Trials) flowchart showing participant flow through allocation to the treatment and waitlist conditions. ISI: Insomnia Severity Index; ITT: intention to treat; NDI: Nightmare Disorder Index; PCL-5: PTSD Checklist for DSM-5; PP: per protocol.

A total of 33 participants responded to the advertisement, registered an account with Federation University’s digital HealthZone platform, and provided baseline data. In total, 9% (3/33) were excluded because they did not meet the selection criteria, and 91% (30/33) were randomized to either the intervention or the waitlist group. A total of 53% (16/30) were randomized into the intervention group, and 47% (14/30) were in the waitlist group (for the participant CONSORT chart, refer to Figure 1). Ages ranged between 18 and 79 years (mean 52.50, SD 16.26 years; N=30), with most participants (19/30, 63%) being female (vs 11/30, 37% male). A total of 67% (20/30) of the participants completed the trial, and their data were analyzed as PP. The ages of the latter group ranged from 18 to 79 years (mean 53.75, SD 16.54 years), with most participants (14/20, 70%) being female (6/20, 30% were male). Demographic variables for the treatment and waitlist groups are shown in Table 2.

**Table 2:** Demographic variables for the treatment and waitlist groups and differences between the 2 at the baseline assessments.

Demographics			Treatment (n=16)		Waitlist (n=14)		Statistic		*P* value^a^
Age (y), mean (SD)			55.56 (15.14)		49.00 (17.34)		t_28_=1.11		.28
**Biological sex, n (%)**							χ^2^_2_=1.4 (N=30)		.51
	Female	11 (69)		8 (57)					
	Male	5 (31)		6 (43)					
**Country, n (%)**							—^b^		—
	Australia	12 (75)		14 (100)					
	Canada	3 (19)		0 (0)					
	United States	1 (6)		0 (0)					
**Educational level, n (%)**							χ^2^_3_=1.2 (N=30)		.75
	Bachelor’s degree	8 (50)		6 (43)					
	Certificate or diploma	4 (25)		6 (43)					
	High school	2 (12)		1 (7)					
	Postgraduate	2 (12)		1 (7)					
**Employment status, n (%)**							χ^2^_4_=2.6 (N=30)		.64
	Employed	7 (44)		5 (36)					
	Looking for work	1 (6)		0 (0)					
	Retired	5 (31)		5 (36)					
	Student	2 (12)		1 (7)					
	Unemployed	1 (6)		3 (21)					
**Relationship status, n (%)**							χ^2^_3_=8.0 (N=30)		.05
	Married	8 (50)		5 (36)					
	Divorced or separated	0 (0)		5 (36)					
	Single	6 (38)		4 (29)					
	Widowed	2 (12)		0 (0)					
**Wildfires experienced, n (%)**							—		—
	2019-2020 wildfires (Australia)	8 (50)		11 (79)					
	Waroona 2016 (Australia)	1 (6)		0 (0)					
	Oregon 2020 (United States)	1 (6)		0 (0)					
	2023 Canadian wildfires	1 (6)		0 (0)					
	2021 BC fires	2 (12)		0 (0)					
	Black Saturday 2009 (Australia)	2 (12)		1 (7)					
	Currowan 2021 (Australia)	1 (6)		0 (0)					
	Sacramento 2014 (United States)	0 (0)		1 (7)					
	Blue Mountains 2013 (Australia)	0 (0)		1 (7)					

^a^Significance level at *P*<.05.

^b^Not applicable.

A total of 4 participants were excluded from the PP analysis for the following reasons: 2 (50%) participants, who completed the pilot trial and provided a complete set of data, revealed that they had restless leg syndrome that was not initially reported during the screening process; 1 (25%) participant received 1 treatment module but withdrew from the study due to illness, providing only baseline measures; and a fourth participant (25%) who provided and completed 50% of the data and modules withdrew due to illness and family commitments. Therefore, the PP analysis was conducted on a sample of 20 participants. In total, 20% (6/30) of the participants provided baseline data; however, they did not initiate treatment and did not respond to emails sent by the research team. Table 1 indicates that there were no significant differences in demographic variables between the intervention and waitlist groups at the baseline assessment (age, *P*=.28; biological sex, *P*=.51; education level, *P*=.75; employment status, *P*=.64; and relationship status, *P*=.05).

To address hypothesis 1, ITT analysis of the mixed-effects regression, including the effect sizes of the DID in the fixed-effects component and the variability in the random-effects component, showed that, at baseline, the waitlist group had mean scores of 3.16, 0.39, and 45.29 on the ISI, NDI, and PCL-5, respectively. At the postintervention time point, the main effect of time, these scores significantly decreased by 1.64 points on the NDI (*P*=.001) and 10.64 points on the PCL-5 (*P*=.009), but no significant (*P*=.06) change was detected on the ISI. The treatment group showed slightly lower baseline scores than the waitlist group, with mean differences of 0.45, 0.04, and 9.20 for the ISI, NDI, and PCL-5, respectively, suggesting that the 2 groups were relatively balanced at baseline. The adjusted DID effects following the treatment indicated that the treatment group experienced greater improvements in mental health measures compared to the waitlist group, with differences of 1.54 points for the ISI, 1.22 points for the NDI, and 4.98 points for the PCL-5. Notably, the improvement in the NDI was marginally significant (*P*=.06), whereas the improvements in the ISI and PCL-5 were not statistically significant (*P*=.26 and *P*=.37, respectively; Table 3).

**Table 3:** Mixed-effects linear regression analysis of the effect of the intervention on primary outcomes (intention-to-treat analysis; N=30)^a^.

Variable		Model 1 (n=60^b^; ISI^c^)		Model 2 (n=60^b^; NDI^d^)		Model 3 (n=60^b^; PCL-5^e^)	
		β (95% CI)	*P* value^f^	β (95% CI)	*P* value^g^	β (95% CI)	*P* value^h^
**Fixed effects**							
	Intercept	3.16 (−0.77 to 7.08)	.12	0.39 (−1.05 to 1.83)	.60	45.29 (37.27 to 53.30)	<.001
**Time**							
	Baseline	Reference	—^i^	Reference	—	Reference	—
	Postintervention time point	−1.88 (−3.86 to 0.10)	.06	−1.64 (−2.58 to −0.71)	.001	−10.64 (−18.65 to −2.64)	.009
**Condition**							
	Waitlist	Reference	—	Reference	—	Reference	—
	Intervention	−0.45 (−2.56 to 1.66)	.68	−0.04 (−1.04 to 0.97)	.95	−9.20 (−20.17 to 1.78)	.10
	Time × condition	−1.54 (−4.26 to 1.17)	.26	−1.22 (−2.50 to 0.06)	.06	−4.98 (−15.94 to 5.98)	.37
**Random effects**							
	SD (time, intercept)	1.41 (0.82 to 2.42)	—	0.82 (0.55 to 1.20)	—	10.82 (7.26 to 16.14)	—
	Correlation (time, intercept)	0.99 (−1.00 to 1.00)	—	1.00 (−1.00 to 1.00)	—	N/A^j^	—
	SD of residual	2.48 (1.86 to 3.31)	—	1.23 (0.86 to 1.47)	—	10.81 (8.39 to 13.92)	—

^a^All models were adjusted for the baseline measure to address the regression-to-mean issue except for model 3, where the baseline measure was excluded for simplicity and convergence.

^b^*n* represents the number of observations in the data with long format to support mixed-effects modeling.

^c^ISI: Insomnia Severity Index.

^d^NDI: Nightmare Disorder Index.

^e^PCL-5: PTSD Checklist for DSM-5.

^f^Model fit—*P*<.001 (Wald chi-square test) and *P*=.03 (likelihood ratio test [LRT]) to compare the mixed-effects models with random-intercept linear models.

^g^Model fit—*P*<.001 (Wald chi-square test) and *P*=.001 (LRT) to compare the mixed-effects models with random-intercept linear models.

^h^Model fit—*P*<.001 (Wald chi-square test) and *P*=.19 (LRT) to compare the mixed-effects models with random-intercept linear models.

^i^Not available.

^j^N/A: not applicable as the random-intercept model better fit the data than the random-intercept, random-slope model. Given its better fit, random-intercept model parameters were reported.

**Figure 2:**
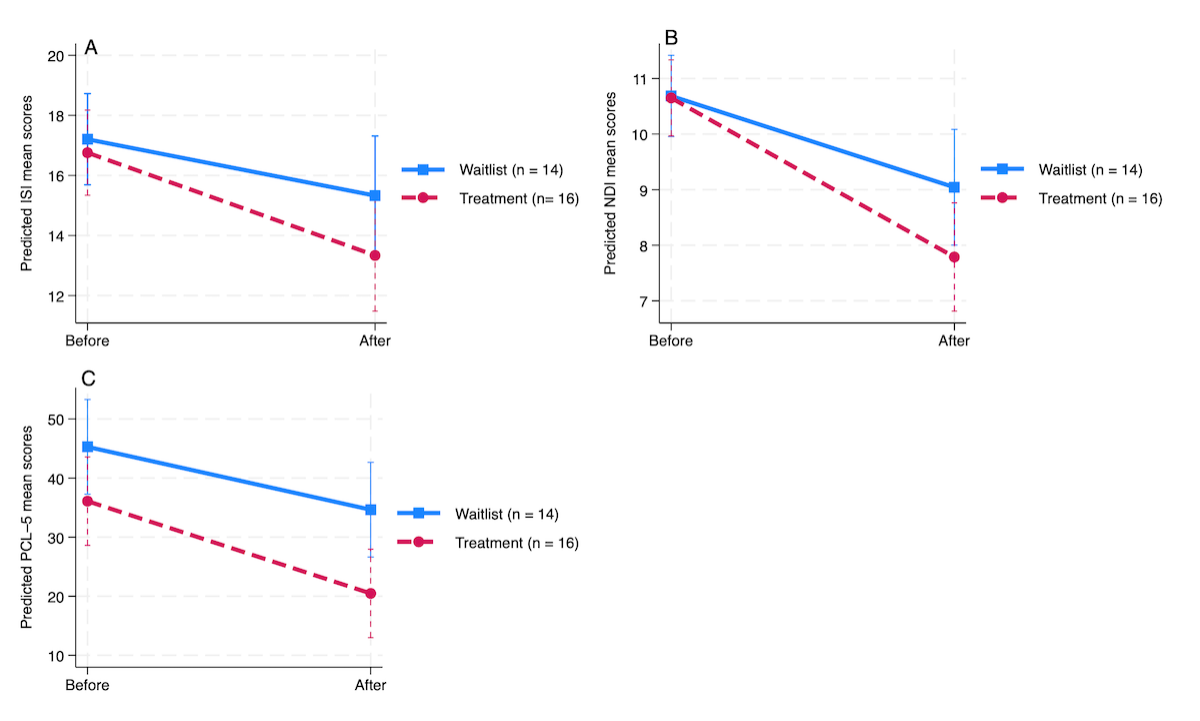
Change in adjusted estimates of primary measures, including the Insomnia Severity Index (ISI); Nightmare Disorder Index (NDI); and PTSD Checklist for DSM-5 (PCL-5), from before to after the intervention based on the intention-to-treat analysis.

In the random-effects part, there was substantial variability among individuals, with average deviations of 1.41, 0.82, and 10.82 points for the ISI, NDI, and PCL-5, respectively, around both the baseline and postintervention means. This suggests considerable individual variability in the outcomes. The positive correlation coefficients between the time point and the intercept indicate that participants with higher baseline scores (above the overall sample mean) were more likely to experience greater decreases in their scores over time compared to those with lower baseline scores. All mixed-effects models for the ISI, NDI, and PCL-5 were statistically significant (*P*<.001). Specifically, the random-intercept, random-slope model was appropriate for the ISI (*P*=.03) and NDI (*P*=.001), whereas a random intercept–only model was sufficient for the PCL-5, which also fit the data but with a nonsignificant *P* value (*P*>.05 and *P*=.19; Table 3). Figure 2 illustrates the changes in primary outcome measures (ISI, NDI, and PCL-5) from before to after treatment based on the ITT analysis.

For completers only, PP analysis of the mixed-effects regression showed that, at baseline, the waitlist group had mean scores of 5.58, 0.46, and 13.37 on the ISI, NDI, and PCL-5, respectively. At the postintervention time point, these scores did not significantly change for the 3 measures (ISI; *P*=.68, NDI; *P*=.24, PCL-5; and *P*=.35). The treatment group baseline scores were balanced compared to those of the waitlist group, with mean differences of 0.99, 0.04, and 3.05 for the ISI, NDI, and PCL-5, respectively. However, the adjusted DID effects indicated an interaction effect of condition × time, with the treatment group experiencing greater and more significant improvements on the NDI with a decrease of 2.27 points (*P*=.049) and a decrease of 13.46 points on the PCL-5 (*P*=.03) but no significant improvements on the ISI (*P*=.25; Table 4).

There was a substantial variability among individuals, with average deviations of 3.41, 1.81, and 9.42 points for the ISI, NDI, and PCL-5, respectively, at the postintervention time point, suggesting considerable individual variability in the outcomes. All mixed-effects models for the ISI, NDI, and PCL-5 were statistically significant (*P*<.001), with a random-intercept model being appropriate for the 3 measures (Table 4).

In relation to the second hypothesis, the ITT analysis showed that, at baseline, the waitlist group had mean scores of 1.66, 12.01, and 0.69 on the GAD-7, PHQ-9, and PSQI, respectively (Table 5). Only the depression scores decreased significantly (*P*<.001) by 6.48 points at the posttreatment time point (main effect of time) in comparison to the waitlist group. The treatment group showed slightly lower baseline scores than those of the waitlist group on the secondary measures, with mean differences of 0.47, 0.90, and 0.04 on the GAD-7, PHQ-9, and PSQI, respectively, suggesting that the 2 groups were balanced at baseline. The adjusted effects at the postintervention time point indicate that the treatment group experienced greater improvements than the waitlist group on all measures but with only significant results on the PSQI (with a difference of 2.22 points; *P*<.001).

**Figure 3:**
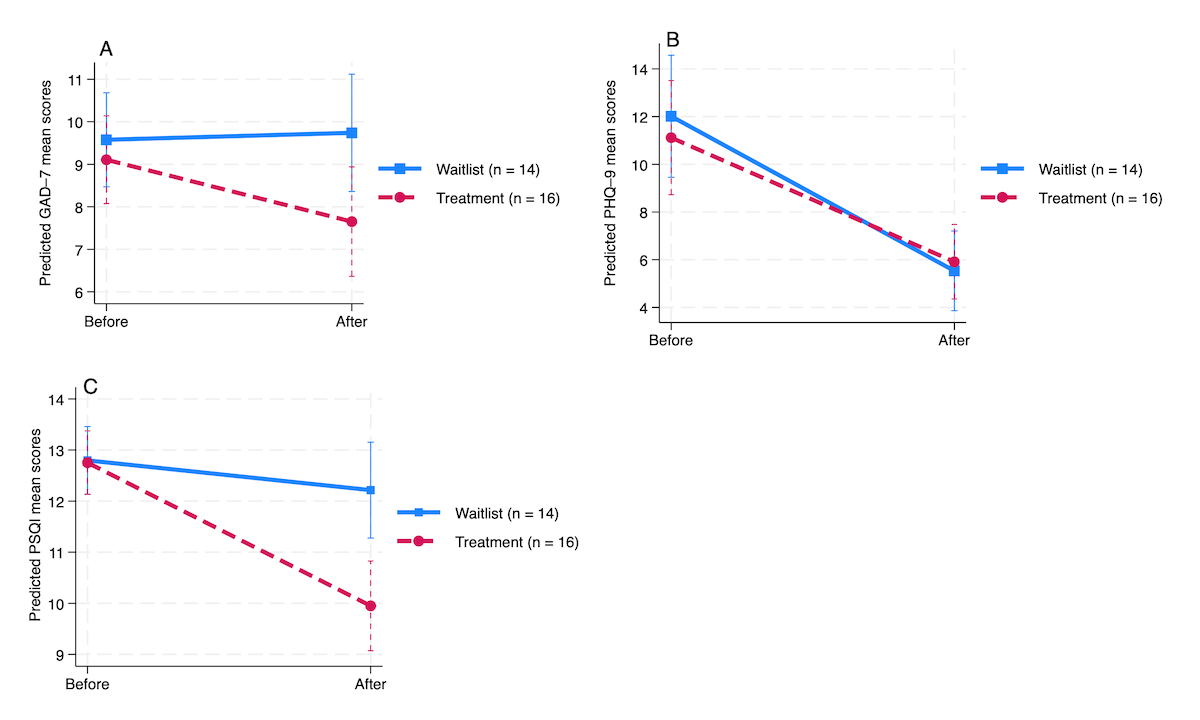
Change in adjusted estimates of secondary outcomes, including the Generalized Anxiety Disorder–7 (GAD-7), Patient Health Questionnaire–9 (PHQ-9), and Pittsburgh Sleep Quality Index (PSQI), from before to after the intervention based on the intention-to-treat analysis.

There was variability among individuals, as shown in Table 5, with average deviations of 0.91, 4.89, and 0.73 points for the GAD-7, PHQ-9, and PSQI, respectively, around both the baseline and postintervention means. The positive correlation coefficients for the GAD-7 and PSQI between the time point and the intercept indicate that participants with higher baseline scores (above the overall sample mean) were more likely to experience greater decrease in their scores over time compared to those with lower baseline scores. Mixed-effects models for the 3 measures were statistically significant (*P*<.001). Specifically, the random-intercept, random-slope model was appropriate for the PHQ-9 and PSQI (*P*=.007 and *P*=.001, respectively), whereas a random intercept–only model was sufficient for the GAD-7 (*P*=.08). Figure 3 shows the difference in decrease in scores between the intervention and waitlist groups for the GAD-7, PHQ-9, and PSQI from the ITT analysis.

For those who completed the study, PP analysis showed that the waitlist mean baseline scores were 11.78, 12.22, and 13.00 on the GAD-7, PHQ-9, and PSQI, respectively. These scores decreased significantly only for the PHQ-9 measure by 6.22 points at the posttreatment time point (*P*<.001). There was also a main effect of condition on the GAD-7 measure with a decrease of 4.14 points (*P*=.04) for the treatment group and an interaction effect of time × condition on the PSQI (*P*=.02), suggesting that the treatment group experienced significantly better sleep quality than the waitlist group following the treatment (Table 6).

Table 6 also shows a great variability among individuals on the random effects, with average deviations of 3.69, 3.16, and 2.82 points for the GAD-7, PHQ-9, and PSQI, respectively, around both the baseline and postintervention means. This suggests substantial individual variability in the outcome measures. Mixed-effects models for the 3 measures were statistically significant (*P*<.001), with the random-intercept, random-slope model being appropriate for the PHQ-9 (*P*=.004), whereas a random intercept–only model was sufficient for the GAD-7 and the PSQI (*P*=.28 and *P*>.99, respectively; Table 6).

To address the third hypothesis, separate mixed-effects linear model analyses were conducted for each group (intervention and waitlist) on all primary and secondary outcome measures. The analysis incorporated data from the following time points: baseline, posttreatment time point, and 3-month follow-up for the intervention group and pretreatment time point, posttreatment time point, and 3-month follow-up for the waitlist group. ITT analysis for the intervention group showed a significant reduction in insomnia, nightmares, and trauma symptoms as measured using the ISI, NDI, and PCL-5 both at the posttreatment time point (*P*<.001) with an effect of 3.42, 2.86, and 15.62 points, respectively, and at the 3-month follow-up (*P*<.001) with an effect of 8.64, 4.20, and 22.83 points, respectively (Table 7).

**Figure 4:**
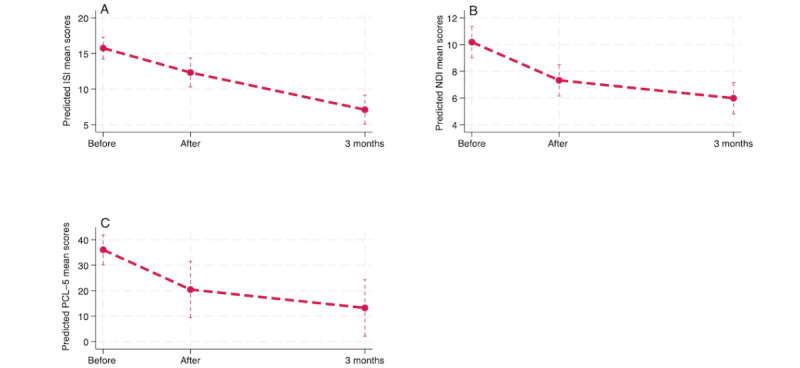
Change in Insomnia Severity Index (ISI); Nightmare Disorder Index (NDI); and PTSD Checklist for DSM-5 (PCL-5) scores for the intervention group from baseline to the postintervention time point and the 3-month follow-up as determined by the intention-to-treat analysis.

A great variability among individuals was detected on the random effects, with average deviations of 1.56, 0.32, and 11.09 points for the ISI, NDI, and PCL-5, respectively, around both the postintervention and 3-month follow-up means. Mixed-effects models for the 3 measures were statistically significant (*P*<.001), with the random-intercept, random-slope model being appropriate for the ISI and the PCL-5 (*P*=007 and *P*=.02, respectively), whereas the random-intercept model was appropriate for the NDI (*P*=.16; Table 7). Figure 4 shows the decrease in scores over time at the postintervention time point and 3-month follow-up for the intervention group.

For those who completed the study, the PP analysis showed comparable results to those reported in the ITT analysis, with a significant reduction in symptoms at both the postintervention time point (ISI; *P*=.04, NDI; *P*<.001, PCL-5; *P*<.001) and 3-month follow-up (*P*<.001 in all cases). The adjusted effects showed a continued reduction in symptoms from the postintervention time point to the 3-month follow-up, with a difference of 7.81, 4.86, and 23.71 points on the ISI, NDI, and PCL-5, respectively (Table 8).

The analysis also showed a substantial variability among individuals, with average deviations of 1.64, 1.03, and 1.81 points for the ISI, NDI, and PCL-5, respectively, around both the baseline and postintervention means. The positive correlation coefficients between the time point and the intercept on the NDI indicate that participants with higher baseline scores (above the overall sample mean) were more likely to experience greater decreases in their scores over time compared to those with lower baseline scores. All mixed-effects models for the ISI, NDI, and PCL-5 were statistically significant (*P*<.001), with a random-intercept model being sufficient for all 3 measures with a nonsignificant *P* value (ISI, *P*=.18; NDI*, P*=.07; and PCL-5, *P*=*.*42, Table 8).

**Figure 5:**
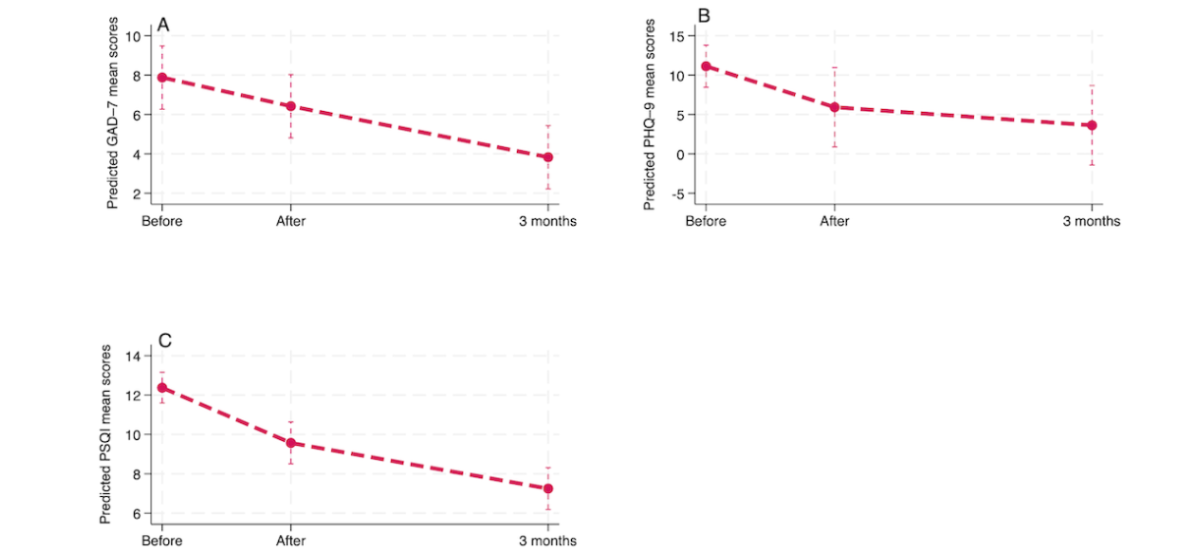
Change in Generalized Anxiety Disorder–7 (GAD-7), Patient Health Questionnaire–9 (PHQ-9), and Pittsburgh Sleep Quality Index (PSQI) scores for the intervention group from baseline to the postintervention time point and the 3-month follow-up as determined by the intention-to-treat analysis.

On the secondary measures, the ITT analysis showed that the intervention group experienced significant improvements on the PHQ-9 and PSQI at both the posttreatment time point and the 3-month follow-up (*P*<.001). No significant reduction in symptoms was observed for the GAD-7 at the posttreatment time point (*P*=.21; Table 9). However, all participants in the intervention group experienced a significant reduction in symptoms at the 3-month follow-up (*P*<.001) on the 3 measures, with adjusted effects showing a decrease of 4.05, 7.50, and 5.12 points on the GAD-7, PHQ-9, and PSQI, respectively. Table 9 also shows variability among individuals on the random effects, with average deviations of 0.001, 5.46, and 0.82 points for the GAD-7, PHQ-9, and PSQI, respectively, around the postintervention and 3-month means. The 3 models were statistically significant (*P*<.001), with the random-intercept, random-slope model being appropriate for the PHQ-9 and PSQI (*P*=.03 and *P*=.002, respectively) and the random-intercept model being appropriate for the GAD-7 (*P*=.34). Figure 5 shows a change in secondary outcomes over time in the intervention group (ITT analysis).

Comparable results were also detected on the PP analysis, with individuals experiencing significant reductions in symptoms following the intervention and also at the 3-month assessments (*P*<.001 in all cases) except for the GAD-7, which was found to be not significant at the postintervention time point (*P*=.29). Participants experienced a greater reduction in symptoms at the 3-month assessment, with adjusted effects indicating a reduction of 3.88, 6.98, and 4.62 points on the GAD-7, PHQ-9, and PSQI, respectively (Table 10). The 3 models were statistically significant (*P*<.001), with the random-intercept model being appropriate for the 3 measures (GAD-7, *P*=.32; PHQ-9, *P*=.56, and PSQI, *P*=.05).

Similarly, ITT analysis results of the mixed-effects regression showed that, at baseline, participants in the waitlist group had mean scores of 10.79, 5.42, and 13.93 on the ISI, NDI, and PCL-5, respectively. The adjusted effects at the postintervention time point indicated that the symptoms significantly reduced by 6.36, 5.78, and 19.37 points, respectively (*P*<.001), and a significant reduction of 8.21, 7.74, and 27.96 points was observed at the 3-month follow-up for the ISI, NDI, and PCL-5, respectively (*P*<.001; Table 11).

**Figure 6:**
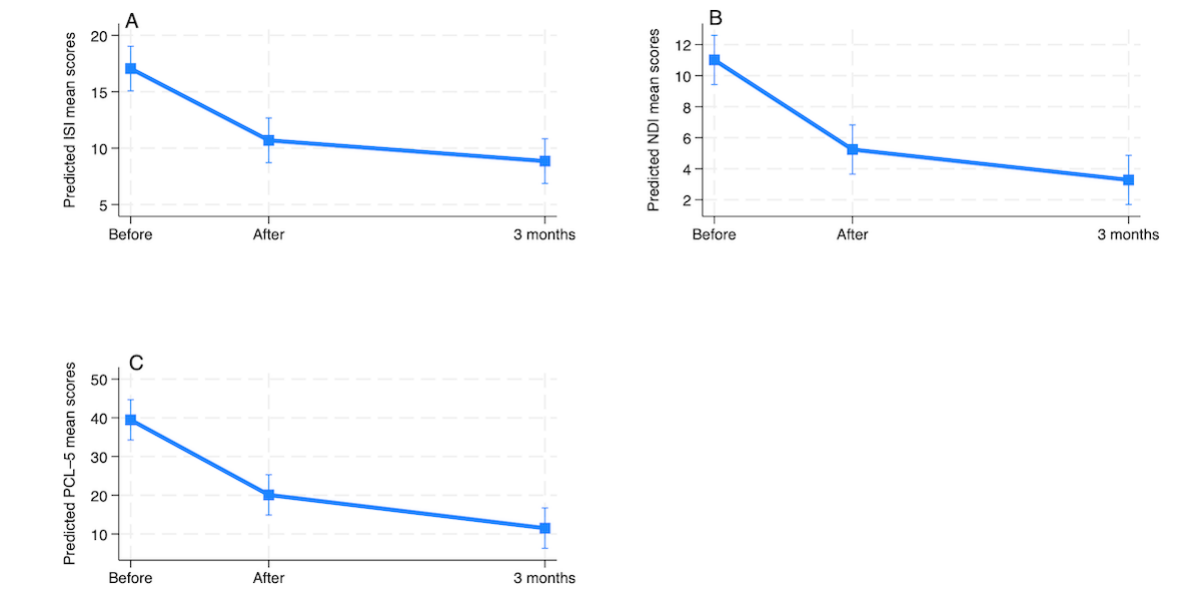
Change in Insomnia Severity Index (ISI); Nightmare Disorder Index (NDI); and PTSD Checklist for DSM-5 (PCL-5) scores for the waitlist group from before to after the intervention and the 3-month follow-up as determined by the intention-to-treat analysis.

In the random-effects part, there was substantial variability among individuals, with average deviations of 9.80, 6.18, and 1.73 points for the ISI, NDI, and PCL-5, respectively, around both the postintervention and 3-month means. This suggests considerable individual variability in the outcomes. All mixed-effects models for the ISI, NDI, and PCL-5 were statistically significant (*P*<.001), with a random-intercept model being appropriate for the 3 measures with a nonsignificant *P* value (*P*>.05; ISI, *P*=.45; NDI, *P*=.59, PCL-5, *P*=14 Table 11). Figure 6 shows the changes in primary outcomes over time for the waitlist group at the postintervention time point and 3-month follow-up.

The PP analysis showed similar results, with significant improvements at both the postintervention and 3-month follow-up assessments on the ISI, NDI, and PCL-5 (*P*<.001 in all cases; Table 12). The mixed-effects models for the ISI, NDI, and PCL-5 were all statistically significant (*P*<.001). The random-intercept model was used for the 3 measures (*P*>.05; ISI, *P*>.99; NDI, *P*>.99; and PCL-5, *P*=.34, Table 12).

For the secondary measures, ITT analysis showed that the waitlist group had baseline mean scores of 0.78, 0.36, and 3.31 on the GAD-7, PHQ-9, and PSQI, respectively. The adjusted effects at the postintervention time point indicated that these scores decreased significantly by 4.72 and 5.07 points for the GAD-7 and the PSQI, respectively (*P*<.001). However, no significant changes were observed on the PHQ-9 at the postintervention time point. Furthermore, adjusted effects showed a significant reduction of 6.51 points on the GAD-7 and 5.00 points on the PSQI (*P*<.001) against the baseline but no changes on the PHQ-9 at the 3-month follow-up (Table 13).

**Figure 7:**
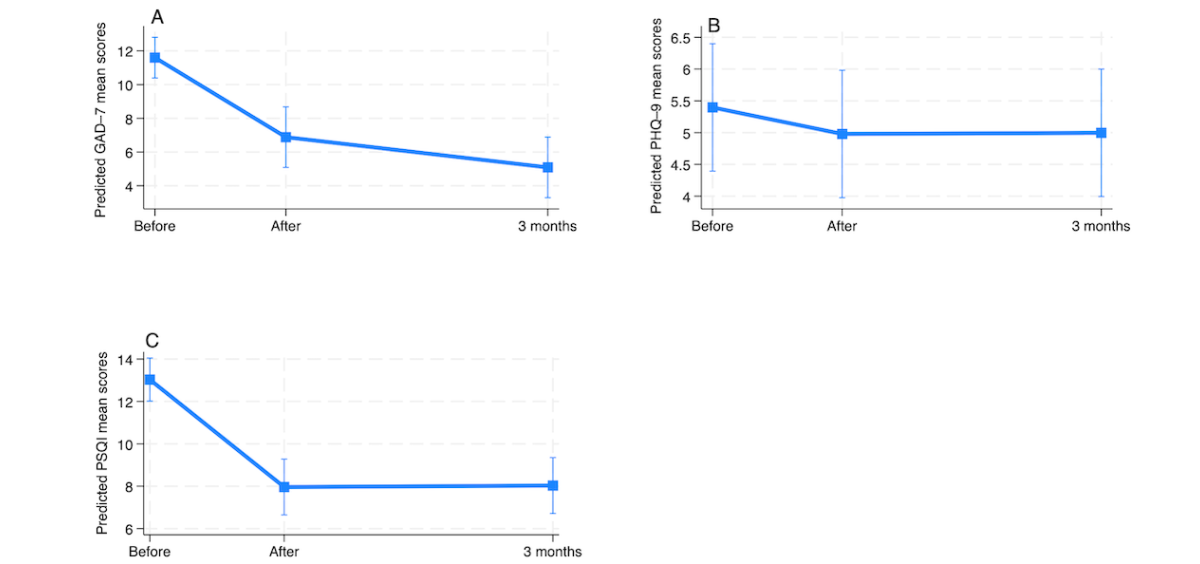
Change in Generalized Anxiety Disorder–7 (GAD-7), Patient Health Questionnaire–9 (PHQ-9), and Pittsburgh Sleep Quality Index (PSQI) scores for the waitlist group from before to after the intervention and the 3-month follow-up as determined by the intention-to-treat analysis.

There was variability among individuals, with deviations of 1.47, 0.06, and 0.92 points for the GAD-7, PHQ-9, and PSQI, respectively, around the postintervention and 3-month follow-up means. The positive correlation coefficients between the time point and the intercept indicate that participants with higher baseline scores (above the overall sample mean) were more likely to experience greater decreases in their scores over time compared to those with lower baseline scores. All mixed-effects models for the GAD-7, PHQ-9, and PSQI were statistically significant (*P*<.001). Specifically, the random-intercept, random-slope model was appropriate for the GAD-7 and PSQI (*P*<.001 and *P*=.02, respectively), whereas a random intercept–only model was sufficient for the PHQ-9, which also fit the data but with a nonsignificant *P* value (*P*>.05; *P*=.17, Table 13). Figure 7 shows the changes in secondary outcomes at the postintervention time point and at the 3-month follow-up for the waitlist group.

For those who completed the study, PP analysis showed similar results for the waitlist group, with significant reduction at the postintervention and 3-month assessments only for the GAD-7 and the PSQI (*P*<.001); however, no significant changes were detected for the PHQ-9 at either the postintervention time point or the 3-month follow-up (*P*=.61 and *P*=.92, respectively; Table 14). For those who experienced significant reductions, there was great variability, with deviations of 1.98 and 1.00 points for the GAD-7 and PSQI, respectively, around the postintervention and 3-month follow-up means. All mixed-effects models for the GAD—7, PHQ—9, and PSQI were statistically significant (*P*<.001). Specifically, the random-intercept model was appropriate for the 3 measures with a nonsignificant *P* values (*P*>.05; GAD-7, *P*=.05, PHQ-9, *P*=.47, PSQI, *P*=.24, Table 14).

**Table 4:** Mixed-effects linear regression analysis of the effect of the intervention on primary outcomes for completers only (per-protocol analysis; N=20)^a^.

Variable		Model 1 (n=40^b^; ISI^c^)			Model 2 (n=40^b^; NDI^d^)			Model 3 (n=40^b^; PCL-5^e^)	
		β (95% CI)	*P* value^f^	β (95% CI)		*P* value^g^	β (95% CI)		*P* value^h^
**Fixed effects**									
	Intercept	5.58 (1.12 to 10.04)	.01	0.46 (−1.70 to 2.61)		.68	13.37 (3.71 to 23.03)		.007
**Time**									
	Baseline	Reference	—^i^	Reference		—	Reference		—
	Postintervention time point	−0.67 (−3.82 to 2.48)	.68	−1.00 (−2.67 to 0.67)		.24	−4.11 (−12.81 to 4.59)		.35
**Condition**									
	Waitlist	Reference	—	Reference		—	Reference		—
	Intervention	−0.99 (−4.06 to 2.04)	.53	−0.04 (−1.65 to 1.56)		.96	−3.05 (−11.52 to 5.42)		.48
	Time × condition	−2.48 (−6.72 to 1.77)	.25	−2.27 (−4.53 to −0.01)		.049	−13.46 (−25.19 to −1.73)		.03
**Random effects**									
	SD (time, intercept)	2.81 (2.60 to 3.22)	—	0.93 (1.31 to 2.00)		—	9.43 (6.67 to 10.33)		—
	Correlation (time, intercept)	0.99 (-1.00 to 1.00)	—	1.00 (-1.00 to 1.00)		—	—		—
	SD of residual	3.41 (2.74 to 4.24)	—	1.81 (1.46 to 2.26)		—	9.42 (7.57 to 11.73)		—

^a^All models were adjusted for the baseline measure to address the regression-to-mean issue.

^b^*n* represents the number of observations in the data with long format to support mixed-effects modeling.

^c^ISI: Insomnia Severity Index.

^d^NDI: Nightmare Disorder Index.

^e^PCL-5: PTSD Checklist for DSM-5.

^f^Model fit—*P*<.001 (Wald chi-square test) and *P*>.99 (likelihood ratio test [LRT]) to compare the mixed-effects models with random-intercept linear models.

^g^Model fit—*P*<.001 (Wald chi-square test) and *P*>.99 (LRT) to compare the mixed-effects models with random-intercept linear models.

^h^Model fit—*P*<.001 (Wald chi-square test) and *P*>.99 (LRT) to compare the mixed-effects models with random-intercept linear models.

^i^Not available.

**Table 5:** Mixed-effects linear regression analysis of the effect of the intervention on secondary outcomes (intention-to-treat analysis; N=30)^a^.

Variable		Model 1 (n=60^b^; GAD-7^c^)		Model 2 (n=60^b^; PHQ-9^d^)		Model 3 (n=60^b^; PSQI^e^)	
		β (95% CI)	*P* value^f^	β (95% CI)	*P* value^g^	β (95% CI)	*P* value^h^
**Fixed effects**							
	Intercept	1.66 (−0.14 to 3.45)	.07	12.01 (9.46 to 14.57)	<.001	0.69 (−1.33 to 2.71)	.50
**Time**							
	Baseline	Reference	—^i^	Reference	—	Reference	—
	Postintervention time point	0.16 (−1.29 to 1.62)	.83	−6.48 (−9.04 to −3.92)	<.001	−0.58 (−1.43 to 0.27)	.18
**Condition**							
	Waitlist	Reference	—	Reference	—	Reference	—
	Intervention	−0.47 (−2.01 to 1.07)	.55	−0.90 (−4.40 to 2.61)	.62	−0.04 (−0.96 to 0.87)	.92
	Time × condition	−1.62 (−3.62 to 0.38)	.11	1.28 (−2.23 to 4.78)	.48	−2.22 (−3.39 to −1.06)	<.001
**Random effects**							
	SD (time and intercept)	0.91 (0.47 to 1.74)	—	4.89 (3.89 to 6.13)	—	0.73 (0.49 to 1.09)	—
	Correlation (time and intercept)	N/A^j^	—	−0.79 (−0.89 to −0.61)	—	0.99 (−1.00 to 1.00)	—
	SD of residual	1.86 (1.39 to 2.50)	—	0.000065 (0.000000665 to 0.01)	—	1.03 (0.78 to 1.34)	—

^a^All models were adjusted for the baseline measure to address the regression-to-mean issue except for model 2, where the baseline measure was excluded for simplicity and convergence.

^b^*n* represents the number of observations in the data with long format to support mixed-effects modeling.

^c^GAD-7: Generalized Anxiety Disorder–7.

^d^PHQ-9: Patient Health Questionnaire–9.

^e^PSQI: Pittsburgh Sleep Quality Index.

^f^Model fit—*P*<.001 (Wald chi-square test) and *P*=.08 (likelihood ratio test [LRT]) to compare the mixed-effects models with random-intercept linear models.

^g^Model fit—*P*<.001 (Wald chi-square test) and *P*=.007 (LRT) to compare the mixed-effects models with random-intercept linear models.

^h^Model fit—*P*<.001 (Wald chi-square test) and *P*=.001 (LRT) to compare the mixed-effects models with random-intercept linear models.

^i^Not available.

^j^N/A: not applicable as the random-intercept model better fit the data than the random-intercept, random-slope model. Given its better fit, random-intercept model parameters were reported.

**Table 6:** Mixed-effects linear regression analysis of the effect of the intervention on secondary outcomes for completers only (per-protocol analysis; N=20)^a^.

Variable		Model 1 (n=40^b^; GAD-7^c^)			Model 2 (n=40^b^; PHQ-9^d^)			Model 3 (n=40^b^; PSQI^e^)	
		β (95% CI)	*P* value^f^	β (95% CI)		*P* value^g^	β (95% CI)		*P* value^h^
**Fixed effects**									
	Intercept	11.78 (8.89 to 14.66)	<.001	12.22 (9.44 to 15.00)		<.001	13.00 (10.87 to 15.13)		<.001
**Time**									
	Baseline	Reference	—^i^	Reference		—	Reference		—
	Postintervention time point	1.33 (−0.90 to 3.57)	.24	−6.22 (−8.86 to −3.59)		<.001	−0.67 (−2.19 to 0.86)		.39
**Condition**									
	Waitlist	Reference	—	Reference		—	Reference		—
	Intervention	−4.14 (−8.03 to −0.25)	.04	−1.60 (−5.35 to 2.15)		.40	−1.00 (−3.88 to 1.88)		.50
	Time × condition	−2.70 (−5.71 to 0.32)	.08	1.14 (−2.41 to 4.70)		.53	−2.42 (−4.48 to −0.37)		.02
**Random effects**									
	SD (intercept)	3.69 (2.52 to 5.41)	—	3.16 (2.01 to 4.98)		—	2.82 (1.95 to 4.07)		—
	Correlation (time, intercept)	N/A^j^	—	−0.68 (−0.85 to −0.37)		—	N/A		—
	SD of residual	2.42 (1.78 to 3.30)	—	2.85 (2.09 to 3.89)		—	1.65 (1.21 to 2.25)		—

^a^All models were adjusted for the baseline measure to address the regression-to-mean issue.

^b^*n* represents the number of observations in the data with long format to support mixed-effects modeling.

^c^GAD-7: Generalized Anxiety Disorder–7.

^d^PHQ-9: Patient Health Questionnaire–9.

^e^PSQI: Pittsburgh Sleep Quality Index.

^f^Model fit—*P*<.001 (Wald chi-square test) and *P*=.28 (likelihood ratio test [LRT]) to compare the mixed-effects models with random-intercept linear models.

^g^Model fit—*P*<.001 (Wald chi-square test) and *P*=.004 (LRT) to compare the mixed-effects models with random-intercept linear models.

^h^Model fit—*P*<.001 (Wald chi-square test) and *P*>.99 (LRT) to compare the mixed-effects models with random-intercept linear models.

^i^Not available.

^j^N/A: not applicable as the random-intercept model better fit the data than the random-intercept, random-slope model. Given its better fit, random-intercept model parameters were reported.

**Table 7:** Mixed-effects linear regression analysis of the change in primary outcomes over time in the intervention group (intention-to-treat analysis; N=16)^a^.

Variable			Model 1 (n=48^b^; ISI^c^)					Model 2 (n=48^b^; NDI^d^)					Model 3 (n=48^b^; PCL-5^e^)		
			β (95% CI)		*P* value^f^		β (95% CI)			*P* value^g^		β (95% CI)			*P* value^h^
**Fixed effects**															
	Intercept	3.23 (−1.66 to 8.12)		.20		3.37 (1.12 to 5.61)			.003		−17.52 (−27.73 to −7.30)			.001	
**Time**															
	Baseline	Reference		—^i^		Reference			—		Reference			—	
	Postintervention time point	−3.42 (−5.47 to −1.38)		.001		−2.86 (−4.51 to −1.21)			.001		−15.62 (−21.87 to −9.37)			<.001	
	3 months after the intervention	−8.64 (−10.68 to −6.60)		<.001		−4.20 (−5.85 to −2.55)			<.001		−22.83 (−29.08 to −16.58)			<.001	
**Random effects**															
	SD (time, intercept)	1.56 (0.91 to 2.67)		—		0.32 (0.0000751 to 0.000133)			—		11.09 (7.06 to 17.43)			—	
	Correlation (time, intercept)	0.99 (−1.00 to 1.00)		—		N/A^j^			—		0.99 (−1.00 to 1.00)			—	
	SD of residual	2.73 (2.11 to 3.55)		—		2.38 (1.86 to 3.04)			—		4.45 (3.36 to 5.89)			—	

^a^All models were adjusted for the baseline measure to address the regression-to-mean issue.

^b^*n* represents the number of observations in the data with long format to support mixed-effects modeling.

^c^ISI: Insomnia Severity Index.

^d^NDI: Nightmare Disorder Index.

^e^PCL-5: PTSD Checklist for DSM-5.

^f^Model fit—*P*<.001 (Wald chi-square test) and *P*=.007 (likelihood ratio test [LRT]) to compare the mixed-effects models with random-intercept linear models.

^g^Model fit—*P*<.001 (Wald chi-square test) and *P*=.16 (LRT) to compare the mixed-effects models with random-intercept linear models.

^h^Model fit—*P*<.001 (Wald chi-square test) and *P*=.02 (LRT) to compare the mixed-effects models with random-intercept linear models.

^i^Not available.

^j^N/A: not applicable as the random-intercept model better fit the data than the random-intercept, random-slope model. Given its better fit, random-intercept model parameters were reported.

**Table 8:** Mixed-effects linear regression analysis of the change in primary outcomes over time in the intervention group for completers only (per-protocol analysis; N=11)^a^.

Variable			Model 1 (n=33^b^; ISI^c^)					Model 2 (n=33^b^; NDI^d^)					Model 3 (n=33^b^; PCL-5^e^)		
			β (95% CI)		*P* value^f^		β (95% CI)			*P* value^g^		β (95% CI)			*P* value^h^
**Fixed effects**															
	Intercept	6.50 (−1.03 to 11.97)		.002		0.24 (−4.19 to 4.66)			.92		16.05 (7.49 to 24.61)			<.001	
**Time**															
	Baseline	Reference		—^i^		Reference			—		Reference			—	
	Postintervention time point	−3.14 (−6.17 to −0.12)		.04		−3.27 (−4.66 to −1.89)			<.001		−17.57 (−25.45 to −9.69)			<.001	
	3 months after the intervention	−7.81 (−10.84 to −4.79)		<.001		−4.86 (−6.24 to −3.47)			<.001		−23.71 (−31.59 to −15.83)			<.001	
**Random effects**															
	SD (intercept)	1.64 (0.49 to 5.44)		—		1.03 (0.24 to 4.48)			—		1.81 (0.01 to 260.12)			—	
	SD of residual	3.62 (2.70 to 4.87)		—		1.49 (0.71 to 3.10)			—		9.43 (7.02 to 12.67)			—	

^a^All models were adjusted for the baseline measure to address the regression-to-mean issue.

^b^*n* represents the number of observations in the data with long format to support mixed-effects modeling.

^c^ISI: Insomnia Severity Index.

^d^NDI: Nightmare Disorder Index.

^e^PCL-5: PTSD Checklist for DSM-5.

^f^Model fit—*P*<.001 (Wald chi-square test) and *P*=.18 (likelihood ratio test [LRT]) to compare the mixed-effects models with random-intercept linear models.

^g^Model fit—*P*<.001 (Wald chi-square test) and *P*=.07 (LRT) to compare the mixed-effects models with random-intercept linear models.

^h^Model fit—*P*<.001 (Wald chi-square test) and *P*=.42 (LRT) to compare the mixed-effects models with random-intercept linear models.

^i^Not available.

**Table 9:** Mixed-effects linear regression analysis of the change in secondary outcomes over time in the intervention group—intention-to-treat analysis (N=16)^a^.

Variable			Model 1 (n=48^b^; GAD-7^c^)					Model 2 (n=48^b^; PHQ-9^d^)					Model 3 (n=48^b^; PSQI^e^)		
			β (95% CI)		*P* value^f^		β (95% CI)			*P* value^g^		β (95% CI)			*P* value^h^
**Fixed effects**															
	Intercept	3.64 (1.58 to 5.70)		.001		−8.64 (−12.69 to −4.59)			.001		0.92 (−2.45 to 4.29)			.59	
**Time**															
	Baseline	Reference		—^i^		Reference			—		Reference			—	
	Postintervention time point	−1.46 (−3.73 to 0.82)		.21		−5.20 (−7.88 to −2.53)			<.001		−2.80 (−3.82 to −1.78)			<.001	
	3 months after the intervention	−4.05 (−6.32 to −1.77)		<.001		−7.50 (−10.17 to −4.82)			<.001		−5.12 (−6.14 to −4.11)			<.001	
**Random effects**															
	SD (time, intercept)	0.001 (0.0000198 to 0.0173)		—		5.46 (4.01 to 7.42)			—		0.82 (0.53 to 1.37)			—	
	Correlation (time, intercept)	N/A^j^		—		0.78 (0.54 to 0.90)			—		0.99 (−1.00 to 1.00)			—	
	SD of residual	3.28 (2.69 to 4.01)		—		0.001 (0.00000549 to 0.20)			—		1.34 (1.04 to 1.73)			—	

^a^All models were adjusted for the baseline measure to address the regression-to-mean issue.

^b^*n* represents the number of observations in the data with long format to support mixed-effects modeling.

^c^GAD-7: Generalized Anxiety Disorder–7.

^d^PHQ-9: Patient Health Questionnaire–9.

^e^PSQI: Pittsburgh Sleep Quality Index.

^f^Model fit—*P*<.001 (Wald chi-square test) and *P*=.34 (likelihood ratio test [LRT]) to compare the mixed-effects models with random-intercept linear models.

^g^Model fit—*P*<.001 (Wald chi-square test) and *P*=.03 (LRT) to compare the mixed-effects models with random-intercept linear models.

^h^Model fit—*P*<.001 (Wald chi-square test) and *P*=.002 (LRT) to compare the mixed-effects models with random-intercept linear models.

^i^Not available.

^j^N/A: not applicable as the random-intercept model better fit the data than the random-intercept, random-slope model. Given its better fit, random-intercept model parameters were reported.

**Table 10:** Mixed-effects linear regression analysis of the change in secondary outcomes over time in the intervention group—per-protocol analysis (N=11)^a^.

Variable		Model 1 (n=33^b^; GAD-7^c^)		Model 2 (n=33^b^; PHQ-9^d^)			Model 3 (n=33^b^; PSQI^e^)	
		β (95% CI)	*P* value^f^	β (95% CI)	*P* value^g^	β (95% CI)		*P* value^h^
**Fixed effects**								
	Intercept	2.85 (0.31 to 5.39)	.03	10.63 (8.28 to 12.98)	<.001	0.85 (−3.05 to 4.76)		.67
**Time**								
	Baseline	Reference	—^i^	Reference	—	Reference		—
	Postintervention time point	−1.36 (−3.86 to 1.14)	.29	−5.08 (−7.80 to −2.36)	<.001	−3.09 (−4.50 to −1.68)		<.001
	3 months after the intervention	−3.88 (−6.38 to −1.38)	.002	−6.98 (−9.71 to −4.26)	<.001	−4.62 (−6.03 to −3.21)		<.001
**Random effects**								
	SD (intercept)	0.90 (0.10 to 8.32)	—	2.28 (1.100 to 4.73)	—	1.12 (0.52 to 2.41)		—
	SD of residual	2.99 (2.23 to 4.02)	—	3.26 (2.42 to 4.38)	—	1.69 (1.26 to 2.27)		—

^a^All models were adjusted for the baseline measure to address the regression-to-mean issue.

^b^*n* represents the number of observations in the data with long format to support mixed-effects modeling.

^c^GAD-7: Generalized Anxiety Disorder–7.

^d^PHQ-9: Patient Health Questionnaire–9.

^e^PSQI: Pittsburgh Sleep Quality Index.

^f^Model fit—*P*<.001 (Wald chi-square test) and *P*=.32 (likelihood ratio test [LRT]) to compare the mixed-effects models with random-intercept linear models.

^g^Model fit—*P*<.001 (Wald chi-square test) and *P*=.56 (LRT) to compare the mixed-effects models with random-intercept linear models.

^h^Model fit—*P*<.001 (Wald chi-square test) and *P*=.05 (LRT) to compare the mixed-effects models with random-intercept linear models.

^i^Not available.

**Table 11:** Mixed-effects linear regression analysis of the change in primary outcomes over time in the waitlist group receiving the intervention—intention-to-treat analysis (N=14)^a^.

Variable			Model 1 (n=42^b^; ISI^c^)				Model 2 (n=42^b^; NDI^d^)					Model 3 (n=42^b^; PCL-5^e^)		
			β (95% CI)		*P* value^f^		β (95% CI)		*P* value^g^		β (95% CI)			*P* value^h^
**Fixed effects**														
	Intercept	10.79 (5.89 to 15.70)		<.001		5.42 (2.74 to 8.10)		<.001		13.93 (4.95 to 22.92)			.002	
**Time**														
	Baseline	Reference		—^i^		Reference		—		Reference			—	
	Postintervention time point	−6.36 (−9.16 to −3.57)		<.001		−5.78 (−8.02 to −3.53)		<.001		−19.37 (−26.62 to −12.13)			<.001	
	3 months after the intervention	−8.21 (−11.01 to −5.41)		<.001		−7.74 (−9.98 to −5.49)		<.001		−27.96 (−35.20 to −20.71)			<.001	
**Random effects**														
	SD (intercept)	0.000098 (−0.21 to 0.21)		—		0.0000618 (−0.14 to 0.14)		—		1.73 (0.01 to 302.24)			—	
	SD of residual	3.78 (3.05 to 4.68)		—		3.03 (2.44 to 3.75)		—		9.78 (7.53 to 12.71)			—	

^a^As the random-intercept model better fit the data than the random-intercept, random-slope model, random-intercept model parameters were reported. All models were adjusted for the pretreatment measure to address the regression-to-mean issue.

^b^*n* represents the number of observations in the data with long format to support mixed-effects modeling.

^c^ISI: Insomnia Severity Index.

^d^NDI: Nightmare Disorder Index.

^e^PCL-5: PTSD Checklist for DSM-5.

^f^Model fit—*P*<.001 (Wald chi-square test) and *P*=.45 (likelihood ratio test [LRT]) to compare the mixed-effects models with random-intercept linear models.

^g^Model fit—*P*<.001 (Wald chi-square test) and *P*=.59 (LRT) to compare the mixed-effects models with random-intercept linear models.

^h^Model fit—*P*<.001 (Wald chi-square test) and *P*=.14 (LRT) to compare the mixed-effects models with random-intercept linear models.

^i^Not available.

**Table 12:** Mixed-effects linear regression analysis of the change in primary outcomes over time in the waitlist group receiving the intervention—per-protocol analysis (N=9)^a^.

Variable		Model 1 (n=27^b^; ISI^c^)		Model 2 (n=27^b^; NDI^d^)			Model 3 (n=27^b^; PCL-5^e^)			
		β (95% CI)	*P* value^f^	β (95% CI)	*P* value^g^	β (95% CI)		*P* value^h^		
**Fixed effects**										
	Intercept	11.65 (5.59 to 17.71)	<.001	5.06 (1.47 to 8.66)	.006	12.85 (−1.41 to 27.11)		.08		
**Time**										
	Baseline	Reference	—^i^	Reference	—	Reference		—		
	Postintervention time point	−6.56 (−10.49 to −2.63)	.001	−5.44 (−8.59 to −2.30)	.001	−20.89 (−30.98 to −10.79)		<.001		
	3 months after the intervention	−7.00 (−10.93 to −3.07)	<.001	−7.11 (−10.26 to −3.96)	<.001	−28.44 (−38.54 to −18.35)		<.001		
**Random effects**										
	SD (intercept)	0.01 (0.01 to 0.6)	—	0.01 (N/A^j^)	—	3.31 (0.29 to 38.15)		—		
	SD of residual	4.25 (3.26 to 5.55)	—	3.41 (2.61 to 4.45)	—	10.93 (7.89 to 15.15)		—		

^a^All models were adjusted for the baseline measure to address the regression-to-mean issue.

^b^*n* represents the number of observations in the data with long format to support mixed-effects modeling.

^c^ISI: Insomnia Severity Index.

^d^NDI: Nightmare Disorder Index.

^e^PCL-5: PTSD Checklist for DSM-5.

^f^Model fit—*P*<.001 (Wald chi-square test) and *P*>.99 (likelihood ratio test [LRT]) to compare the mixed-effects models with random-intercept linear models.

^g^Model fit—*P*<.001 (Wald chi-square test) and *P*>.99 (LRT) to compare the mixed-effects models with random-intercept linear models.

^h^Model fit—*P*<.001 (Wald chi-square test) and *P*=.34 (LRT) to compare the mixed-effects models with random-intercept linear models.

^i^Not available.

^j^N/A: not applicable.

**Table 13:** Mixed-effects linear regression analysis of the change in secondary outcomes over time in the waitlist group receiving the intervention—intention-to-treat analysis (N=14)^a^.

Variable			Model 1 (n=42^b^; GAD-7^c^)					Model 2 (n=42^b^; PHQ-9^d^)					Model 3 (n=42^b^; PSQI^e^)		
			β (95% CI)		*P* value^f^		β (95% CI)			*P* value^g^		β (95% CI)			*P* value^h^
**Fixed effects**															
	Intercept	0.78 (−2.68 to 4.24)		.66		0.36 (−1.00 to 1.72)			.61		3.31 (–0.62 to 7.25)			.10	
**Time**															
	Baseline	Reference		—^i^		Reference			—		Reference			—	
	Postintervention time point	−4.72 (−6.24 to −3.19)		<.001		−0.42 (−1.84 to 1.00)			.56		−5.07 (−6.42 to −3.71)			<.001	
	3 months after the intervention	−6.51 (−8.03 to −4.99)		<.001		−0.40 (−1.82 to 1.02)			.58		−5.00 (−6.35 to −3.65)			<.001	
**Random effects**															
	SD (time, intercept)	1.47 (0.87 to 2.48)		—		0.06 (2.72 × 10^–64^ to 1.3 × 10^61^)			—		0.92 (0.48 to 1.77)			—	
	Correlation (time, intercept)	0.99 (−1.00 to 1.00)		—		N/A^j^			—		0.99 (−1.00 to 1.00)			—	
	SD of residual	1.78 (1.33 to 2.37)		—		1.91 (1.48 to 2.48)			—		1.71 (1.28 to 2.78)			—	

^a^All models were adjusted for the baseline measure to address the regression-to-mean issue.

^b^*n* represents the number of observations in the data with long format to support mixed-effects modeling.

^c^GAD-7: Generalized Anxiety Disorder–7.

^d^PHQ-9: Patient Health Questionnaire–9.

^e^PSQI: Pittsburgh Sleep Quality Index.

^f^Model fit—*P*<.001 (Wald chi-square test) and *P*<.001 (likelihood ratio test [LRT]) to compare the mixed-effects models with random-intercept linear models.

^g^Model fit—*P*<.001 (Wald chi-square test) and *P*=.17 (LRT) to compare the mixed-effects models with random-intercept linear models.

^h^Model fit—*P*<.001 (Wald chi-square test) and *P*=.02 (LRT) to compare the mixed-effects models with random-intercept linear models.

^i^Not available.

^j^N/A: not applicable as the random-intercept model better fit the data than the random-intercept, random-slope model. Given its better fit, random-intercept model parameters were reported.

**Table 14:** Mixed-effects linear regression analysis of the change in secondary outcomes over time in the waitlist group receiving the intervention—per-protocol analysis (N=9)^a^.

Variable		Model 1 (n=27^b^; GAD-7^c^)			Model 2 (n=27^b^; PHQ-9^d^)			Model 3 (n=27^b^; PSQI^e^)	
		β (95% CI)	*P* value^f^	β (95% CI)		*P* value^g^	β (95% CI)		*P* value^h^
**Fixed effects**									
	Intercept	3.85 (−8.29 to −3.04)	.22	0.43 (−1.61 to 2.48)		.68	5.89 (1.55 to 10.22)		.008
**Time**									
	Baseline	Reference	—^i^	Reference		—	Reference		—
	Postintervention time point	−5.67 (−8.29 to −3.04)	<.001	−0.56 (−2.69 to 1.58)		.61	−4.78 (−7.05 to −2.50)		<.001
	3 months after the intervention	−7.78 (−10.40 to −5.15)	<.001	−0.11 (−2.25 to 2.03)		.92	−4.78 (−7.05 to −2.50)		<.001
**Random effects**									
	SD (intercept)	1.98 (0.88 to 4.46)	—	0.29 (0.0000012 to 69,857.52)		—	1.00 (0.21 to 4.67)		—
	SD of residual	2.84 (2.05 to 3.94)	—	2.31 (1.67 to 3.21)		—	2.46 (1.78 to 3.41)		—

^a^All models were adjusted for the baseline measure to address the regression-to-mean issue.

^b^*n* represents the number of observations in the data with long format to support mixed-effects modeling.

^c^GAD-7: Generalized Anxiety Disorder–7.

^d^PHQ-9: Patient Health Questionnaire–9.

^e^PSQI: Pittsburgh Sleep Quality Index.

^f^Model fit—*P*<.001 (Wald chi-square test) and *P*=.05 (likelihood ratio test [LRT]) to compare the mixed-effects models with random-intercept linear models.

^g^Model fit—*P*<.001 (Wald chi-square test) and *P*=.47 (LRT) to compare the mixed-effects models with random-intercept linear models.

^h^Model fit—*P*<.001 (Wald chi-square test) and *P*=.24 (LRT) to compare the mixed-effects models with random-intercept linear models.

^i^Not available.

### Clinical Significance

Table 15 presents the number of participants achieving clinical significance from baseline to 3-month follow-up, based on intention-to-treat (ITT) analysis. The results show that a higher percentage of waitlist group participants achieved clinically significant changes in nightmare symptoms (mean change 7.74, SD 5.12) and PTSD symptoms (mean difference 27.96, SD 16.56) compared to the treatment group (mean change 4.20, SD 4.21 for nightmare symptoms and mean change: 22.83, SD 17.09 for PTSD symptoms). In contrast, a higher percentage of treatment group participants achieved clinically significant changes in insomnia symptoms (mean difference 8.65, SD 5.28) compared to the waitlist group (mean change 8.21, SD 6.96).

Two participants were missing 3-month follow-up data on the ISI, NDI, and PCL-5.

The per-protocol (PP) analysis (Table 16) yielded similar results to the ITT analysis. Specifically, a higher percentage of waitlist group participants achieved clinically significant changes in nightmare symptoms (mean change 7.11, SD 5.71) and PTSD symptoms (mean change 28.44, SD 18.58) compared to the treatment group (mean change 4.67, SD 3.04 for nightmare symptoms and mean change: 24.84, SD 18.62 for PTSD symptoms). Conversely, a higher percentage of treatment group participants achieved clinically significant changes in insomnia symptoms (mean change 7.33, SD 5.87) compared to the waitlist group (mean change 7.00, SD 8.34). However, the differences between the two groups were not statistically significant.

**Table 15:** Distribution of clinically significant responders by condition—intention-to-treat analysis (N=30).

Outcome	Waitlist (n=14), n (%)	Treatment (n=16), n (%)	*P* value
ISI^a^	8 (57)	10 (62)	.77
NDI^b^	13 (93)	10 (62)	.05
PCL-5^c^	11 (79)	9 (56)	.20

^a^ISI: Insomnia Severity Index.

^b^NDI: Nightmare Disorder Index.

^c^PCL-5: PTSD Checklist for DSM-5.

**Table 16:** Distribution of clinically significant responders by condition—per-protocol analysis (N=18) (N=20).

Outcome	Waitlist (n=9), n (%)	Treatment (n=9), n (%)	*P* value
ISI^a^	4 (44)	5 (56)	.64
NDI^b^	8 (89)	5 (56)	.11
PCL-5^c^	7 (78)	4 (44)	.15

^a^ISI: Insomnia Severity Index.

^b^NDI: Nightmare Disorder Index.

^c^PCL-5: PTSD Checklist for DSM-5.

### Satisfaction and Engagement With Treatment

Participants were asked to rate how likely they were to revisit the treatment modules on a 5-point, single-item Likert scale. A frequency analysis showed that most participants responded with *strongly agree* (9/20, 45%) and *moderately agree* (7/20, 35%). In total, 10% (2/20) of the participants responded with *moderately disagree*, and 10% (2/20) of the participants did not respond to this question. The number of log-ins to the site was observed as an indicator of the level of engagement by participants with the modules, and it was found that the 20 participants who were included in the PP analysis had an average of 8.5 log-ins during their engagement with Sleep Best-i. A total of 5% (2/20) logged in 12 times to the site; 5% (3/20) logged in 5 times; 5% (2/20) logged in 7 times; 10% (3/20) logged in 6 times; 15% (4/20) logged in 11 times; and 20% (6/20) logged in 8, 9, and 10 times. On average, participants spent 116 minutes on the site visiting modules and completing assessments.

## Discussion

### Principal Findings

The aim of this clinical pilot trial was to assess the feasibility of a brief (6 modules over 4 weeks), self-paced, digital intervention for the treatment of insomnia, nightmares, and PTSD symptoms in an international sample of survivors of wildfires. The first hypothesis was partially supported. The PP analysis revealed a significant interaction effect of condition × time on both the NDI and the PCL-5, indicating that Sleep Best-i effectively reduced symptoms of nightmares and PTSD from before to after the intervention in the treatment group compared to the waitlist group. However, no significant changes were observed in insomnia symptoms. The ITT analysis yielded similar findings, with a significant main effect of time showing a reduction in nightmare and PTSD symptoms at the posttreatment time point for the intervention group but no significant changes in insomnia symptoms. In examining the 2 groups separately, Sleep Best-i significantly reduced symptoms of insomnia, nightmares, and PTSD from baseline to the postintervention time point, and this improvement in symptoms was maintained at the 3-month assessment for the 2 groups across both the PP and ITT analyses. This study’s findings diverge from those of previous research using CBTi to treat insomnia in survivors of wildfires [27,28] and veterans [25,26]. This discrepancy may be attributed to the brief duration of insomnia treatment in our study, which spanned only the first 2 weeks, unlike in other clinical trials that used CBTi in ≥6 sessions. Research suggests that an average of 6 to 8 sessions is typically required to significantly reduce insomnia symptoms [13,64]. The shorter session duration used in this study may have contributed to the differing outcomes. Nevertheless, the maintenance of improvements at the 3-month follow-up is a promising indicator of the intervention’s long-term effectiveness.

These findings further substantiate the efficacy of ERRT in reducing the severity and frequency of nightmares, aligning with those of previous studies [36,65-67]. These results are also consistent with those of other clinical trials demonstrating that CBTi and ERRT can lead to significant reductions in PTSD symptoms at the posttreatment time point [36,68]. Notably, it is possible that symptoms of trauma and nightmares are more malleable and responsive to treatment, whereas insomnia symptoms may be more entrenched and less amenable to change with brief interventions, suggesting a potential explanation for the observed differences in treatment outcomes.

In relation to the second hypothesis, the PP analysis revealed that Sleep Best-i not only alleviated symptoms of nightmares and trauma but also significantly reduced anxiety symptoms (main effect of condition) in the treatment group compared to the waitlist group at the postintervention time point. In addition, the treatment group experienced a significant reduction in depressive symptoms at the posttreatment time point (main effect of time) and a significant interaction effect of time × condition on sleep quality, indicating more pronounced improvements in sleep quality at the postintervention time point compared to the waitlist group. The ITT analysis yielded similar findings, with significant reductions in depressive symptoms at the postintervention time point and a significant interaction effect of time × condition on sleep quality. However, the reduction in anxiety symptoms was no longer significant in the ITT analysis. This is notable as research suggests a strong relationship between insomnia and anxiety symptoms, with the expectation that successful insomnia treatment would lead to a reduction in anxiety symptoms [69].

When analyzing the 2 groups separately, both the PP and ITT analyses yielded similar results, with few exceptions. Notably, both groups showed significant reductions in anxiety symptoms at the 3-month follow-up, although this improvement was only sustained for the waitlist group at the postintervention assessment. In terms of sleep quality, both groups showed significant improvements at both the postintervention and 3-month follow-up assessments. However, only the intervention group exhibited significant reductions in depressive symptoms at both the postintervention and 3-month follow-up assessments.

This study’s findings align with those of existing research demonstrating the effectiveness of CBTi-based treatments for comorbid conditions [13,70]. While part of this study’s findings corroborates those of previous studies showing a significant reduction in symptoms of depression following CBT treatment for insomnia [27,28], the lack of improvement in symptoms of depression in the waitlist group is not well understood. One possible explanation is that the insignificant improvements in insomnia symptoms may have contributed to the absence of change in depression levels [69]. In addition, external life difficulties may have influenced symptoms of depression during the study period. Notably, the 2 groups had distinct intervention experiences, which may have impacted the outcomes. Specifically, the 3-month follow-up assessment for the waitlist group occurred 4 weeks after the treatment group’s follow-up, potentially introducing passage-of-time effects that may have influenced the results. This study’s findings are also in line with those of other studies that found improved sleep quality on the PSQI following the administration of CBTi [71].

In terms of clinical significance, a substantial proportion of participants achieved MCSC (according to the PP analysis) in insomnia (9/18, 50%), nightmares (13/18, 72%), and PTSD symptoms (11/18, 61%). Notably, the MCSC rates for insomnia and PTSD in this study were slightly higher than those reported in previous clinical trials using CBTi and imagery rehearsal therapy. For example, Ulmer et al [26] found that 55.4% and 50% of their sample (N=22) achieved clinical significance in insomnia and PTSD, respectively. In contrast, the MCSC rates in this study were lower than those reported by Belleville et al [27], who found that 64.7% and 70.6% of participants achieved MCSC for insomnia and PTSD at the posttreatment time point, respectively, and 64.7% and 58.8% achieved MCSC for insomnia and PTSD at the 3-month follow-up, respectively. The discrepancy in MCSC rates between our study and others may be attributed to the shorter treatment duration of Sleep Best-i (4 weeks) than those of the other trials (6-12 weeks) [26,27].

A significant proportion of participants expressed a high level of satisfaction with the intervention, with approximately 80% (18/20) of users reporting a positive experience. Furthermore, the level of engagement by participants with the modules was measured using the number of log-ins into the site. The 20 participants included in the PP analysis had an average of 8.5 log-in times during their engagement with Sleep Best-i. The log-in frequency was varied, with 5% (2/20) of the participants logging in 12 times; 5% (3/20) logging in 5 times; 5% (2/20) logging in 7 times; 10% (3/20) logging in 6 times; 15% (4/20) logging in 11 times; and 20% (6/20) logging in 8, 9, or 10 times. On average, participants spent 116 minutes on the site visiting modules and completing assessments.

### Limitations

This clinical pilot trial has limitations that warrant consideration. The sample consisted of self-selected individuals, and the absence of clinical assessments to confirm diagnoses of insomnia, nightmares, and PTSD introduces a potential source of bias. The small sample size is another impediment, underscoring the need for further testing with a larger and more diverse population to establish the external validity of the intervention. One major concern in relation to Sleep Best-i is the PTSD module and its potential for triggering trauma symptoms. Although this risk was anticipated, participants were provided with emergency numbers. Moreover, throughout the initial 2-week treatment period, participants received training in cognitive and behavioral strategies to effectively manage stress. It is important to note that our study was conducted as an open-label trial. Future research should prioritize addressing the identified gaps and limitations, thereby enhancing the validity and generalizability of the findings in this field.

### Future Directions

This feasibility trial provides valuable insights into the effectiveness of the Sleep Best-i intervention, a CBT-based program for survivors of wildfires. While our findings are promising, we recognize that future research could further enhance our understanding of its efficacy and effectiveness. One potential direction for future research could involve stratifying the assessment of outcomes based on the type of survivor by dividing the population into subgroups based on specific characteristics, such as demographic characteristics such as age, sex, and income; life experiences, including previous trauma and social support; experiences with wildfires, such as severity of exposure and proximity to the fire; losses experienced as a result of wildfires, including property damage and loss of loved ones; social support from family, friends, and community resources; and institutional support provided during the recovery phase, including access to mental health services and financial assistance. By doing so, researchers can identify patterns and trends within each subgroup, compare outcomes between subgroups, and develop targeted interventions that address the unique needs of each subgroup. In addition, ecological assessments over longer time frames could provide a more comprehensive understanding of the intervention’s impact in real-world settings. We propose that future studies consider these approaches to build on our findings and inform the development of more targeted and effective interventions for survivors of wildfires.

### Conclusions

Taken together, these findings indicate that Sleep Best-i incorporating CBTi and ERRT improved nightmares, PTSD, sleep quality, and symptoms of depression from baseline to the posttreatment time point. This positive impact was sustained at the 3-month follow-up for the 2 groups, with some variations on anxiety and depression. Participants in the intervention group, when assessed separately, experienced improvements on all measures from before to after treatment and at the 3-month follow-up, with the exception of anxiety symptoms at the posttreatment time point. The waitlist group experienced a significant reduction in symptoms on all measures from before treatment to after treatment and at the 3-month follow-up except for symptoms of depression. This clinical trial is the first in the field of sleep disturbances to use a concise, digital, self-paced intervention over 4 weeks among survivors of wildfires.

## Appendix

Multimedia Appendix 1 CONSORT-eHEALTH checklist (V 1.6.1).

Multimedia Appendix 2 Plain-language statement.

Multimedia Appendix 3 Consent form.

## Abbreviations

CBT: cognitive behavioral therapy
CBTi: cognitive behavioral therapy for insomnia
CONSORT: Consolidated Standards of Reporting Trials
CONSORT-EHEALTH: Consolidated Standards of Reporting Trials of Electronic and Mobile Health Applications and Online Telehealth
DID: difference-in-differences
DSM-5: Diagnostic and Statistical Manual of Mental Disorders, Fifth Edition
ERRT: exposure, relaxation, and rescripting therapy
GAD-7: Generalized Anxiety Disorder–7
ISI: Insomnia Severity Index
ITT: intention-to-treat
MCSC: minimal clinically significant change
NDI: Nightmare Disorder Index
PCL-5: PTSD Checklist for DSM-5
PHQ-9: Patient Health Questionnaire–9
PLIS: plain-language information statement
PP: per protocol
PSQI: Pittsburgh Sleep Quality Index
PTSD: posttraumatic stress disorder

## Data Availability

The datasets generated or analyzed during this study are not publicly available due to ethical restrictions, but are available from the corresponding author on reasonable request.
