# Supplementary material for: Digital Cognitive Behavioral Therapy–Based Treatment for Insomnia, Nightmares, and Posttraumatic Stress Disorder Symptoms in Survivors of Wildfires: Pilot Randomized Feasibility Trial
Source: JMIR Hum Factors. 2025 Mar 14;12:e65228. doi: 10.2196/65228 (PMC11953604; doi:10.2196/65228)
Supplement: Multimedia Appendix 1 [file humanfactors_v12i1e65228_app1.pdf]

CONSORT-EHEALTH (V 1.6.1) - Submission/Publication Form

The CONSORT-EHEALTH checklist is intended for authors of randomized trials evaluating web-based and Internet-based applications/interventions, including mobile interventions, electronic games (incl multiplayer games), social media, certain telehealth applications, and other interactive and/or networked electronic applications. Some of the items (e.g. all subitems under item 5 - description of the intervention) may also be applicable for other study designs.

The goal of the CONSORT EHEALTH checklist and guideline is to be
a) a guide for reporting for authors of RCTs,
b) to form a basis for appraisal of an ehealth trial (in terms of validity)

CONSORT-EHEALTH items/subitems are MANDATORY reporting items for studies published in the Journal of Medical Internet Research and other journals / scientific societies endorsing the checklist.

Items numbered 1., 2., 3., 4a., 4b etc are original CONSORT or CONSORT-NPT (non-pharmacologic treatment) items.
Items with Roman numerals (i., ii, iii, iv etc.) are CONSORT-EHEALTH extensions/clarifications.

As the CONSORT-EHEALTH checklist is still considered in a formative stage, we would ask that you also RATE ON A SCALE OF 1-5 how important/useful you feel each item is FOR THE PURPOSE OF THE CHECKLIST and reporting guideline (optional).

Mandatory reporting items are marked with a red *.
In the textboxes, either copy & paste the relevant sections from your manuscript into this form - please include any quotes from your manuscript in QUOTATION MARKS,
or answer directly by providing additional information not in the manuscript, or elaborating on why the item was not relevant for this study.

YOUR ANSWERS WILL BE PUBLISHED AS A SUPPLEMENTARY FILE TO YOUR PUBLICATION IN JMIR AND ARE CONSIDERED PART OF YOUR PUBLICATION (IF ACCEPTED).
Please fill in these questions diligently. Information will not be copyedited, so please use proper spelling and grammar, use correct capitalization, and avoid abbreviations.

DO NOT FORGET TO SAVE AS PDF _AND_ CLICK THE SUBMIT BUTTON SO YOUR ANSWERS ARE IN OUR DATABASE !!!

Citation Suggestion (if you append the pdf as Appendix we suggest to cite this paper in the caption):
Eysenbach G, CONSORT-EHEALTH Group
CONSORT-EHEALTH: Improving and Standardizing Evaluation Reports of Web-based and Mobile Health Interventions
J Med Internet Res 2011;13(4):e126

URL: http://www.jmir.org/2011/4/e126/
doi: 10.2196/jmir.1923
PMID: 22209829

Sign in to Google to save your progress. Learn more

* Indicates required question

Your name *

First Last

Fadia Isaac

Primary Affiliation (short), City, Country *

University of Toronto, Toronto, Canada

Federation University, Victoria, Australia

Your e-mail address *

abc@gmail.com

fisaac@students.federation.edu.au

Title of your manuscript *

Provide the (draft) title of your manuscript.

Digital cognitive-behavioral therapy-based treatment for insomnia, nightmares and post-traumatic stress disorder symptoms in wildfire survivors: A randomized feasibility pilot trial

Name of your App/Software/Intervention *

If there is a short and a long/alternate name, write the short name first and add the long name in brackets.

Sleep Best-i

Evaluated Version (if any)

e.g. "V1", "Release 2017-03-01", "Version 2.0.27913"

No version

Language(s) *

What language is the intervention/app in? If multiple languages are available, separate by comma (e.g. "English, French")

English

URL of your Intervention Website or App

e.g. a direct link to the mobile app on app in appstore (itunes, Google Play), or URL of the website. If the intervention is a DVD or hardware, you can also link to an Amazon page.

https://www.youtube.com/watch?v=iN_aSJk3iTY

URL of an image/screenshot (optional)

Your answer

Accessibility *

Can an enduser access the intervention presently?

- ○ access is free and open
- ◉ access only for special usergroups, not open
- ○ access is open to everyone, but requires payment/subscription/in-app purchases
- ○ app/intervention no longer accessible
- ○ Other:

Primary Medical Indication/Disease/Condition *

e.g. "Stress", "Diabetes", or define the target group in brackets after the condition, e.g. "Autism (Parents of children with)", "Alzheimers (Informal Caregivers of)"

Sleep and trauma symptoms in (wildfire surviv

Primary Outcomes measured in trial *

comma-separated list of primary outcomes reported in the trial

Insomnia Severity Index (ISI), Nightmare Disor[illegible]

Secondary/other outcomes

Are there any other outcomes the intervention is expected to affect?

Generalized Anxiety Disorder Questionnaire (GAD-7), Patient Health Questionnaire (PHQ-9), and the Pittsburgh Sleep Quality Index (PSQI)

Recommended "Dose" *

What do the instructions for users say on how often the app should be used?

- ( ) Approximately Daily
- (x) Approximately Weekly
- ( ) Approximately Monthly
- ( ) Approximately Yearly
- ( ) "as needed"
- ( ) Other:

Approx. Percentage of Users (starters) still using the app as recommended after 3 months *

- (x) unknown / not evaluated
- ( ) 0-10%
- ( ) 11-20%
- ( ) 21-30%
- ( ) 31-40%
- ( ) 41-50%
- ( ) 51-60%
- ( ) 61-70%
- ( ) 71%-80%
- ( ) 81-90%
- ( ) 91-100%
- ( ) Other:

Overall, was the app/intervention effective? *

- ( ) yes: all primary outcomes were significantly better in intervention group vs control
- (•) partly: SOME primary outcomes were significantly better in intervention group vs control
- ( ) no statistically significant difference between control and intervention
- ( ) potentially harmful: control was significantly better than intervention in one or more outcomes
- ( ) inconclusive: more research is needed
- ( ) Other:

Article Preparation Status/Stage *

At which stage in your article preparation are you currently (at the time you fill in this form)

- (•) not submitted yet - in early draft status
- ( ) not submitted yet - in late draft status, just before submission
- ( ) submitted to a journal but not reviewed yet
- ( ) submitted to a journal and after receiving initial reviewer comments
- ( ) submitted to a journal and accepted, but not published yet
- ( ) published
- ( ) Other:

Journal *

If you already know where you will submit this paper (or if it is already submitted), please provide the journal name (if it is not JMIR, provide the journal name under "other")

- ○ not submitted yet / unclear where I will submit this
- ○ Journal of Medical Internet Research (JMIR)
- ○ JMIR mHealth and UHealth
- ○ JMIR Serious Games
- ◉ JMIR Mental Health
- ○ JMIR Public Health
- ○ JMIR Formative Research
- ○ Other JMIR sister journal
- ○ Other:

Is this a full powered effectiveness trial or a pilot/feasibility trial? *

- ◉ Pilot/feasibility
- ○ Fully powered

Manuscript tracking number *

If this is a JMIR submission, please provide the manuscript tracking number under "other" (The ms tracking number can be found in the submission acknowledgement email, or when you login as author in JMIR. If the paper is already published in JMIR, then the ms tracking number is the four-digit number at the end of the DOI, to be found at the bottom of each published article in JMIR)

- ○ no ms number (yet) / not (yet) submitted to / published in JMIR
- ◉ Other: 65228

TITLE AND ABSTRACT

1a) TITLE: Identification as a randomized trial in the title

1a) Does your paper address CONSORT item 1a? *

I.e does the title contain the phrase "Randomized Controlled Trial"? (if not, explain the reason under "other")

- (●) yes
- ( ) Other:

1a-i) Identify the mode of delivery in the title

Identify the mode of delivery. Preferably use “web-based” and/or “mobile” and/or “electronic game” in the title. Avoid ambiguous terms like “online”, “virtual”, “interactive”. Use “Internet-based” only if Intervention includes non-web-based Internet components (e.g. email), use “computer-based” or “electronic” only if offline products are used. Use “virtual” only in the context of “virtual reality” (3-D worlds). Use “online” only in the context of “online support groups”. Complement or substitute product names with broader terms for the class of products (such as “mobile” or “smart phone” instead of “iphone”), especially if the application runs on different platforms.

	1	2	3	4	5	
subitem not at all important	( )	( )	( )	( )	(●)	essential

Clear selection

Does your paper address subitem 1a-i? *

Copy and paste relevant sections from manuscript title (include quotes in quotation marks "like this" to indicate direct quotes from your manuscript), or elaborate on this item by providing additional information not in the ms, or briefly explain why the item is not applicable/relevant for your study

"Digital cognitive-behavioral therapy-based treatment for insomnia, nightmares and post-traumatic stress disorder symptoms in wildfire survivors: A randomized feasibility pilot trial"

1a-ii) Non-web-based components or important co-interventions in title

Mention non-web-based components or important co-interventions in title, if any (e.g., “with telephone support”).

	1	2	3	4	5	
subitem not at all important	◉	○	○	○	○	essential

Clear selection

Does your paper address subitem 1a-ii?

Copy and paste relevant sections from manuscript title (include quotes in quotation marks "like this" to indicate direct quotes from your manuscript), or elaborate on this item by providing additional information not in the ms, or briefly explain why the item is not applicable/relevant for your study

Your answer

1a-iii) Primary condition or target group in the title

Mention primary condition or target group in the title, if any (e.g., “for children with Type I Diabetes”) Example: A Web-based and Mobile Intervention with Telephone Support for Children with Type I Diabetes: Randomized Controlled Trial

	1	2	3	4	5	
subitem not at all important	○	○	○	○	◉	essential

Clear selection

Does your paper address subitem 1a-iii? *

Copy and paste relevant sections from manuscript title (include quotes in quotation marks "like this" to indicate direct quotes from your manuscript), or elaborate on this item by providing additional information not in the ms, or briefly explain why the item is not applicable/relevant for your study

Digital cognitive-behavioral therapy-based treatment for "insomnia, nightmares and post-traumatic stress disorder symptoms in wildfire survivors": A randomized feasibility pilot trial

1b) ABSTRACT: Structured summary of trial design, methods, results, and conclusions

NPT extension: Description of experimental treatment, comparator, care providers, centers, and blinding status.

1b-i) Key features/functionalities/components of the intervention and comparator in the METHODS section of the ABSTRACT

Mention key features/functionalities/components of the intervention and comparator in the abstract. If possible, also mention theories and principles used for designing the site. Keep in mind the needs of systematic reviewers and indexers by including important synonyms. (Note: Only report in the abstract what the main paper is reporting. If this information is missing from the main body of text, consider adding it)

	1	2	3	4	5	
subitem not at all important	○	○	○	○	◉	essential

Clear selection

Does your paper address subitem 1b-i? *

Copy and paste relevant sections from the manuscript abstract (include quotes in quotation marks "like this" to indicate direct quotes from your manuscript), or elaborate on this item by providing additional information not in the ms, or briefly explain why the item is not applicable/relevant for your study

"This pilot trial evaluated the feasibility of a four-week, digital, self-paced intervention combining cognitive behavioral therapy for insomnia (CBTi), and exposure, relaxation, and rescripting therapy for nightmares (ERRT) in wildfire survivors from Australia, Canada and the United States of America".

1b-ii) Level of human involvement in the METHODS section of the ABSTRACT

Clarify the level of human involvement in the abstract, e.g., use phrases like “fully automated” vs. “therapist/nurse/care provider/physician-assisted” (mention number and expertise of providers involved, if any). (Note: Only report in the abstract what the main paper is reporting. If this information is missing from the main body of text, consider adding it)

	1	2	3	4	5	
subitem not at all important	○	○	○	◉	○	essential

Clear selection

Does your paper address subitem 1b-ii?

Copy and paste relevant sections from the manuscript abstract (include quotes in quotation marks "like this" to indicate direct quotes from your manuscript), or elaborate on this item by providing additional information not in the ms, or briefly explain why the item is not applicable/relevant for your study

This pilot trial evaluated the feasibility of a four-week, digital, "self-paced intervention "

1b-iii) Open vs. closed, web-based (self-assessment) vs. face-to-face assessments in the METHODS section of the ABSTRACT

Mention how participants were recruited (online vs. offline), e.g., from an open access website or from a clinic or a closed online user group (closed usergroup trial), and clarify if this was a purely web-based trial, or there were face-to-face components (as part of the intervention or for assessment). Clearly say if outcomes were self-assessed through questionnaires (as common in web-based trials). Note: In traditional offline trials, an open trial (open-label trial) is a type of clinical trial in which both the researchers and participants know which treatment is being administered. To avoid confusion, use “blinded” or “unblinded” to indicated the level of blinding instead of “open”, as “open” in web-based trials usually refers to “open access” (i.e. participants can self-enrol). (Note: Only report in the abstract what the main paper is reporting. If this information is missing from the main body of text, consider adding it)

	1	2	3	4	5	
subitem not at all important	○	○	○	○	◉	essential

Clear selection

Does your paper address subitem 1b-iii?

Copy and paste relevant sections from the manuscript abstract (include quotes in quotation marks "like this" to indicate direct quotes from your manuscript), or elaborate on this item by providing additional information not in the ms, or briefly explain why the item is not applicable/relevant for your study

"Study participants were recruited between May and December 2023 through social media platforms, workshops, conferences, and radio interviews". "They were informed about the purpose, allocation, and the structure of the study". "Participants also completed self-report secondary outcome measures".

1b-iv) RESULTS section in abstract must contain use data

Report number of participants enrolled/assessed in each group, the use/uptake of the intervention (e.g., attrition/adherence metrics, use over time, number of logins etc.), in addition to primary/secondary outcomes. (Note: Only report in the abstract what the main paper is reporting. If this information is missing from the main body of text, consider adding it)

	1	2	3	4	5	
subitem not at all important	○	○	○	○	◉	essential

Clear selection

Does your paper address subitem 1b-iv?

Copy and paste relevant sections from the manuscript abstract (include quotes in quotation marks "like this" to indicate direct quotes from your manuscript), or elaborate on this item by providing additional information not in the ms, or briefly explain why the item is not applicable/relevant for your study

"Thirty wildfire survivors were allocated to either the treatment group (n=16) or the waitlist control group (n=14) in a sequential manner. Participants' ages ranged from 18 to 79 years, with a mean age of 52.5 years (SD = 16.26). The cohort consisted of 19 females (63.3%) and 11 males (36.7%)".

1b-v) CONCLUSIONS/DISCUSSION in abstract for negative trials

Conclusions/Discussions in abstract for negative trials: Discuss the primary outcome - if the trial is negative (primary outcome not changed), and the intervention was not used, discuss whether negative results are attributable to lack of uptake and discuss reasons. (Note: Only report in the abstract what the main paper is reporting. If this information is missing from the main body of text, consider adding it)

	1	2	3	4	5	
subitem not at all important	(selected)	○	○	○	○	essential

Clear selection

Does your paper address subitem 1b-v?

Copy and paste relevant sections from the manuscript abstract (include quotes in quotation marks "like this" to indicate direct quotes from your manuscript), or elaborate on this item by providing additional information not in the ms, or briefly explain why the item is not applicable/relevant for your study

Your answer

INTRODUCTION

2a) In INTRODUCTION: Scientific background and explanation of rationale

2a-i) Problem and the type of system/solution

Describe the problem and the type of system/solution that is object of the study: intended as stand-alone intervention vs. incorporated in broader health care program? Intended for a particular patient population? Goals of the intervention, e.g., being more cost-effective to other interventions, replace or complement other solutions? (Note: Details about the intervention are provided in “Methods” under 5)

	1	2	3	4	5	
subitem not at all important	○	○	○	○	◉	essential

Clear selection

Does your paper address subitem 2a-i? *

Copy and paste relevant sections from the manuscript (include quotes in quotation marks "like this" to indicate direct quotes from your manuscript), or elaborate on this item by providing additional information not in the ms, or briefly explain why the item is not applicable/relevant for your study

Research on trauma and sleep disorders has predominantly centered on conducting clinical trials for treatment among military veterans, with a significant body of work focused on this population [19-21]. There is a need to focus on developing treatments for people who have experienced the trauma of natural disasters including wildfires. The main objective of this pilot trial was to assess the feasibility of a brief, digital, and self-paced intervention called Sleep Best-i in alleviating symptoms of insomnia, nightmares, and PTSD among survivors of wildfires. A second objective was to compare the severity of symptom reduction between the intervention and a waitlist condition.

2a-ii) Scientific background, rationale: What is known about the (type of) system

Scientific background, rationale: What is known about the (type of) system that is the object of the study (be sure to discuss the use of similar systems for other conditions/diagnoses, if appropiate), motivation for the study, i.e. what are the reasons for and what is the context for this specific study, from which stakeholder viewpoint is the study performed, potential impact of findings [2]. Briefly justify the choice of the comparator.

	1	2	3	4	5	
subitem not at all important	○	○	○	○	◉	essential

Clear selection

Does your paper address subitem 2a-ii? *

Copy and paste relevant sections from the manuscript (include quotes in quotation marks "like this" to indicate direct quotes from your manuscript), or elaborate on this item by providing additional information not in the ms, or briefly explain why the item is not applicable/relevant for your study

There have only been two clinical trials addressing the treatment of sleep disturbances and PTSD in wildfire survivors [22,23]. Both trials showed that the application of sleep dynamic therapy and CBTi therapist-assisted self-help interventions led to significant reduction in insomnia and PTSD symptoms following treatment.
While studies support the effectiveness of CBT, CBTi and ERRT in treating trauma symptoms, insomnia and nightmares, accessing these treatments particularly when wildfires cause destructions to infrastructure can be challenging. This, in turn, causes difficulty in the delivery of counselling services to remote locations where large numbers of people are affected and are in great need. Digital therapies can bridge this gap by delivering evidence-based treatments in communities affected by the trauma of wildfires [24,25]. However, the feasibility of digital therapies should be firstly examined in clinical trials before they are operationalised in the field [22,26].

2b) In INTRODUCTION: Specific objectives or hypotheses

Does your paper address CONSORT subitem 2b? *

Copy and paste relevant sections from the manuscript (include quotes in quotation marks "like this" to indicate direct quotes from your manuscript), or elaborate on this item by providing additional information not in the ms, or briefly explain why the item is not applicable/relevant for your study

The study tested the following hypotheses: (1) Sleep Best-i will result in significant reductions in insomnia, nightmares, and PTSD symptoms from baseline to post-treatment, compared to the waitlist group, (2) Sleep Best-i will also lead to significant reductions in anxiety and depression scores, and improvements in sleep quality, from baseline to post-treatment, compared to the waitlist group, (3) both groups (within group effects) will experience significant improvements in all measures (insomnia, nightmares, PTSD symptoms, anxiety, depression, and sleep quality) from baseline to post-treatment, and these improvements will be maintained at the 3-month follow-up after receiving Sleep Best-i.

METHODS

3a) Description of trial design (such as parallel, factorial) including allocation ratio

Does your paper address CONSORT subitem 3a? *

Copy and paste relevant sections from the manuscript (include quotes in quotation marks "like this" to indicate direct quotes from your manuscript), or elaborate on this item by providing additional information not in the ms, or briefly explain why the item is not applicable/relevant for your study

This study used a parallel arm, sequential alternation method of assigning participants to either intervention or waitlist groups in a 1:1 ratio, with a crossover of the latter group. The alternation method ensures the ability to answer questions about the effectiveness of treatments to inform decision making in clinical practice [27].

3b) Important changes to methods after trial commencement (such as eligibility criteria), with reasons

Does your paper address CONSORT subitem 3b? *

Copy and paste relevant sections from the manuscript (include quotes in quotation marks "like this" to indicate direct quotes from your manuscript), or elaborate on this item by providing additional information not in the ms, or briefly explain why the item is not applicable/relevant for your study

Since its initiation, the protocol has undergone only minor revisions, specifically an update to the statistical analysis methodology to include linear mixed methods and ITT analysis.

3b-i) Bug fixes, Downtimes, Content Changes

Bug fixes, Downtimes, Content Changes: ehealth systems are often dynamic systems. A description of changes to methods therefore also includes important changes made on the intervention or comparator during the trial (e.g., major bug fixes or changes in the functionality or content) (5-iii) and other "unexpected events" that may have influenced study design such as staff changes, system failures/downtimes, etc. [2].

	1	2	3	4	5	
subitem not at all important	○	○	◉	○	○	essential

Clear selection

Does your paper address subitem 3b-i?

Copy and paste relevant sections from the manuscript (include quotes in quotation marks "like this" to indicate direct quotes from your manuscript), or elaborate on this item by providing additional information not in the ms, or briefly explain why the item is not applicable/relevant for your study

HealthZone site was monitored daily during the trial to track any potential concerns or issues associate with the site or the participants.

4a) Eligibility criteria for participants

Does your paper address CONSORT subitem 4a? *

Copy and paste relevant sections from the manuscript (include quotes in quotation marks "like this" to indicate direct quotes from your manuscript), or elaborate on this item by providing additional information not in the ms, or briefly explain why the item is not applicable/relevant for your study

To be eligible to take part in the trial, participants had to: (1) provide a digital consent; (2) score ≥ 8 on the Insomnia Severity Index (ISI): (3) and/or score ≥ 3 on the Nightmare Disorder Index (NDI): (4) and/or score ≥ 31 on the PTSD CheckList – Civilian Version (PCL-5); (5) be fluent in the English language; (6) and have access to the internet. Exclusion criteria included the following; not a wildfire survivor, diagnosis of psychotic disorder, diagnosis of sleep apnoea and/or restless leg syndrome, current use of steroids for any health condition, diagnosis of alcohol or drug dependence, and attending psychotherapy for either sleep and/or PTSD conditions.

4a-i) Computer / Internet literacy

Computer / Internet literacy is often an implicit “de facto” eligibility criterion - this should be explicitly clarified.

	1	2	3	4	5	
subitem not at all important	○	○	○	○	◉	essential

Clear selection

Does your paper address subitem 4a-i?

Copy and paste relevant sections from the manuscript (include quotes in quotation marks "like this" to indicate direct quotes from your manuscript), or elaborate on this item by providing additional information not in the ms, or briefly explain why the item is not applicable/relevant for your study

"5) be fluent in the English language"

4a-ii) Open vs. closed, web-based vs. face-to-face assessments:

Open vs. closed, web-based vs. face-to-face assessments: Mention how participants were recruited (online vs. offline), e.g., from an open access website or from a clinic, and clarify if this was a purely web-based trial, or there were face-to-face components (as part of the intervention or for assessment), i.e., to what degree got the study team to know the participant. In online-only trials, clarify if participants were quasi-anonymous and whether having multiple identities was possible or whether technical or logistical measures (e.g., cookies, email confirmation, phone calls) were used to detect/prevent these.

	1	2	3	4	5	
subitem not at all important	○	○	○	○	◉	essential

Clear selection

Does your paper address subitem 4a-ii? *

Copy and paste relevant sections from the manuscript (include quotes in quotation marks "like this" to indicate direct quotes from your manuscript), or elaborate on this item by providing additional information not in the ms, or briefly explain why the item is not applicable/relevant for your study

"Participants were informed that their participation was voluntary. To ensure the secure storage and handling of collected data, it was stored on a password-protected platform (HealthZone) accessible only to the researchers involved in the study. To protect participant confidentiality, names were replaced with unique identifiers, and only country of residence was collected, with no addresses recorded. The data will be retained for a period of five years before being destroyed".

"Participants were recruited between May 2023 and December 2023 via social media platforms including international Facebook campaigns, radio broadcasts, LinkedIn, Reddit, and online community noticeboards".

"Participants were informed about the purpose, allocation, and the structure of the study as explained in the PLIS. However, they were not informed about the study's hypotheses".

"Wildfire survivors who were interested read a PLIS, signed a digital consent to register an account on Federation University's digital platform/ HealthZone and provided baseline data by completing demographics as well as self-report measures on insomnia, nightmares and trauma symptoms".

"Once participants gained access to their personal dashboard within HealthZone, they were able to access information about the trial, complete the self-report secondary measures and access the treatment modules consecutively".

4a-iii) Information giving during recruitment

Information given during recruitment. Specify how participants were briefed for recruitment and in the informed consent procedures (e.g., publish the informed consent documentation as appendix, see also item X26), as this information may have an effect on user self-selection, user expectation and may also bias results.

	1	2	3	4	5	
subitem not at all important	○	○	○	○	◉	essential

Clear selection

Does your paper address subitem 4a-iii?

Copy and paste relevant sections from the manuscript (include quotes in quotation marks "like this" to indicate direct quotes from your manuscript), or elaborate on this item by providing additional information not in the ms, or briefly explain why the item is not applicable/relevant for your study

The below paragraph has been taken from the PLIS that has also been included in the appendix. we have also included the consent form in the appendix.

"Potential participants will answer online questions that are likely to take 5-10 minutes in duration. The questions include demographic information, questions about wildfires/bushfires, current medications, mental health history, questions that assess sleeping difficulties, trauma symptoms and questions about nightmares. Eligible participants will have to sign a consent form. Those signing the consent form will complete other online questions that will take 5 minutes to complete. The online questions assess symptoms of depression, anxiety and sleep quality. Participants will also be offered the opportunity to attend a clinical interview with a clinical psychologist to establish diagnosis of sleep disorders should they wish. The clinical interview, 15 minutes in duration, will ask questions about sleep difficulties to establish diagnosis of sleep disorders, trauma symptoms, and questions about medications. The clinical psychologist running the clinical interviews, has 13 years of experience and has received training in diagnosing and treating sleep and trauma symptoms. Eligible participants will be assigned to either an intervention group or a waitlist group. The intervention group will receive treatment immediately and the waitlist group will start receiving treatment after 4 weeks"
.

4b) Settings and locations where the data were collected

Does your paper address CONSORT subitem 4b? *

Copy and paste relevant sections from the manuscript (include quotes in quotation marks "like this" to indicate direct quotes from your manuscript), or elaborate on this item by providing additional information not in the ms, or briefly explain why the item is not applicable/relevant for your study

"To ensure the secure storage and handling of collected data, it was stored on a password-protected platform (HealthZone) accessible only to the researchers involved in the study. To protect participant confidentiality, names were replaced with unique identifiers, and only country of residence was collected, with no addresses recorded. The data will be retained for a period of five years before being destroyed".

4b-i) Report if outcomes were (self-)assessed through online questionnaires

Clearly report if outcomes were (self-)assessed through online questionnaires (as common in web-based trials) or otherwise.

	1	2	3	4	5	
subitem not at all important	○	○	○	○	◉	essential

Clear selection

Does your paper address subitem 4b-i? *

Copy and paste relevant sections from the manuscript (include quotes in quotation marks "like this" to indicate direct quotes from your manuscript), or elaborate on this item by providing additional information not in the ms, or briefly explain why the item is not applicable/relevant for your study

'Wildfire survivors who were interested read a PLIS, signed a digital consent to register an account on Federation University's digital platform/ HealthZone and provided baseline data by completing demographics as well as self-report measures on insomnia, nightmares and trauma symptoms"

"Once participants gained access to their personal dashboard within HealthZone, they were able to access information about the trial, complete the self-report secondary measures and access the treatment modules consecutively"

4b-ii) Report how institutional affiliations are displayed

Report how institutional affiliations are displayed to potential participants [on ehealth media], as affiliations with prestigious hospitals or universities may affect volunteer rates, use, and reactions with regards to an intervention.(Not a required item – describe only if this may bias results)

	1	2	3	4	5	
subitem not at all important	○	○	◉	○	○	essential

Clear selection

Does your paper address subitem 4b-ii?

Copy and paste relevant sections from the manuscript (include quotes in quotation marks "like this" to indicate direct quotes from your manuscript), or elaborate on this item by providing additional information not in the ms, or briefly explain why the item is not applicable/relevant for your study

"An advertisement about the pilot study with a URL link was distributed on social media platforms displaying Federation University and the Natural Hazards Research Australia affiliation".

5) The interventions for each group with sufficient details to allow replication, including how and when they were actually administered

5-i) Mention names, credential, affiliations of the developers, sponsors, and owners

Mention names, credential, affiliations of the developers, sponsors, and owners [6] (if authors/evaluators are owners or developer of the software, this needs to be declared in a "Conflict of interest" section or mentioned elsewhere in the manuscript).

	1	2	3	4	5	
subitem not at all important	○	○	○	○	◉	essential

Clear selection

Does your paper address subitem 5-i?

Copy and paste relevant sections from the manuscript (include quotes in quotation marks "like this" to indicate direct quotes from your manuscript), or elaborate on this item by providing additional information not in the ms, or briefly explain why the item is not applicable/relevant for your study

"Sleep Best-i was specifically designed for this clinical trial by some researchers of this study (FI, BK & GK)".

5-ii) Describe the history/development process

Describe the history/development process of the application and previous formative evaluations (e.g., focus groups, usability testing), as these will have an impact on adoption/use rates and help with interpreting results.

	1	2	3	4	5	
subitem not at all important	○	○	○	○	◉	essential

Clear selection

Does your paper address subitem 5-ii?

Copy and paste relevant sections from the manuscript (include quotes in quotation marks "like this" to indicate direct quotes from your manuscript), or elaborate on this item by providing additional information not in the ms, or briefly explain why the item is not applicable/relevant for your study

This is a feasibility study. Details about the digital program were given as follows:

"The collated treatment manual draws from evidence-based treatment manuals authored by other sleep researchers [14,16,30-34]. The main therapeutic methods used in the manual were CBTi and ERRT. The recorded digital modules featured human recorded voice and animated videos utilizing VideoScribe [35] that explained concepts related to sleep and trauma symptoms, as well as provided instructions on implementing treatment. The intervention also offered two role plays of therapeutic sessions to demonstrate cognitive restructuring, dream rescripting, and sleep scheduling. Sleep Best-i consisted of six modules administered over a four-week period".

5-iii) Revisions and updating

Revisions and updating. Clearly mention the date and/or version number of the application/intervention (and comparator, if applicable) evaluated, or describe whether the intervention underwent major changes during the evaluation process, or whether the development and/or content was "frozen" during the trial. Describe dynamic components such as news feeds or changing content which may have an impact on the replicability of the intervention (for unexpected events see item 3b).

	1	2	3	4	5	
subitem not at all important	○	○	◉	○	○	essential

Clear selection

Does your paper address subitem 5-iii?

Copy and paste relevant sections from the manuscript (include quotes in quotation marks "like this" to indicate direct quotes from your manuscript), or elaborate on this item by providing additional information not in the ms, or briefly explain why the item is not applicable/relevant for your study

"Once released, the modules remained unchanged throughout the trial, and the intervention was successfully implemented without encountering technical difficulties".

5-iv) Quality assurance methods

Provide information on quality assurance methods to ensure accuracy and quality of information provided [1], if applicable.

	1	2	3	4	5	
subitem not at all important	○	○	◉	○	○	essential

Clear selection

Does your paper address subitem 5-iv?

Copy and paste relevant sections from the manuscript (include quotes in quotation marks "like this" to indicate direct quotes from your manuscript), or elaborate on this item by providing additional information not in the ms, or briefly explain why the item is not applicable/relevant for your study

"There were periodical checks to ensure modules were compliant with the treatment manual".
"HealthZone site was monitored daily during the trial to track any potential concerns or issues associate with the site or the participants".

5-v) Ensure replicability by publishing the source code, and/or providing screenshots/screen-capture video, and/or providing flowcharts of the algorithms used

Ensure replicability by publishing the source code, and/or providing screenshots/screen-capture video, and/or providing flowcharts of the algorithms used. Replicability (i.e., other researchers should in principle be able to replicate the study) is a hallmark of scientific reporting.

	1	2	3	4	5	
subitem not at all important	◉	○	○	○	○	essential

Clear selection

Does your paper address subitem 5-v?

Copy and paste relevant sections from the manuscript (include quotes in quotation marks "like this" to indicate direct quotes from your manuscript), or elaborate on this item by providing additional information not in the ms, or briefly explain why the item is not applicable/relevant for your study

Due to copyright restrictions, the program's materials and content are not publicly available for publication, dissemination, or reproduction. Access to the program has only been provided to reviewers.

5-vi) Digital preservation

Digital preservation: Provide the URL of the application, but as the intervention is likely to change or disappear over the course of the years; also make sure the intervention is archived (Internet Archive, webcitation.org, and/or publishing the source code or screenshots/videos alongside the article). As pages behind login screens cannot be archived, consider creating demo pages which are accessible without login.

	1	2	3	4	5	
subitem not at all important	○	○	○	◉	○	essential

Clear selection

Does your paper address subitem 5-vi?

Copy and paste relevant sections from the manuscript (include quotes in quotation marks "like this" to indicate direct quotes from your manuscript), or elaborate on this item by providing additional information not in the ms, or briefly explain why the item is not applicable/relevant for your study

"All links have been archived in Wayback Machine".

5-vii) Access

Access: Describe how participants accessed the application, in what setting/context, if they had to pay (or were paid) or not, whether they had to be a member of specific group. If known, describe how participants obtained “access to the platform and Internet” [1]. To ensure access for editors/reviewers/readers, consider to provide a “backdoor” login account or demo mode for reviewers/readers to explore the application (also important for archiving purposes, see vi).

	1	2	3	4	5	
subitem not at all important	○	○	○	○	◉	essential

Clear selection

Does your paper address subitem 5-vii? *

Copy and paste relevant sections from the manuscript (include quotes in quotation marks "like this" to indicate direct quotes from your manuscript), or elaborate on this item by providing additional information not in the ms, or briefly explain why the item is not applicable/relevant for your study

"The modules were available to participants from the start of the trial to the end the follow up period. Participants were able to access their dashboard up to four weeks following the 3-months follow-up data collection".

"As a gesture of appreciation, participants who completed the study were offered an Aus $100 e-voucher (approximately USD $66.70)".

"Once participants gained access to their personal dashboard within HealthZone, they were able to access information about the trial, complete the self-report secondary measures and access the treatment modules consecutively. Participants were able to access the treatment modules at no cost via either a mobile phone, a computer or an iPad".

5-viii) Mode of delivery, features/functionalities/components of the intervention and comparator, and the theoretical framework

Describe mode of delivery, features/functionalities/components of the intervention and comparator, and the theoretical framework [6] used to design them (instructional strategy [1], behaviour change techniques, persuasive features, etc., see e.g., [7, 8] for terminology). This includes an in-depth description of the content (including where it is coming from and who developed it) [1]," whether [and how] it is tailored to individual circumstances and allows users to track their progress and receive feedback" [6]. This also includes a description of communication delivery channels and – if computer-mediated communication is a component – whether communication was synchronous or asynchronous [6]. It also includes information on presentation strategies [1], including page design principles, average amount of text on pages, presence of hyperlinks to other resources, etc. [1].

	1	2	3	4	5	
subitem not at all important	○	○	○	○	◉	essential

Clear selection

Does your paper address subitem 5-viii? *

Copy and paste relevant sections from the manuscript (include quotes in quotation marks "like this" to indicate direct quotes from your manuscript), or elaborate on this item by providing additional information not in the ms, or briefly explain why the item is not applicable/relevant for your study

"Participants had the option of providing a pseudo name if they wished. However, they needed to provide a valid email address to allow communication about the study. Their email addresses were also directly connected to their personal dashboard on HealthZone. The baseline data were viewed by two researchers to decide eligibility. Those who were eligible (assessed by FI and GK, both clinical psychologists) were contacted via email to notify them of their eligibility, their randomization and giving them instructions on how to access their personal dashboard on HealthZone".

"The modules were released sequentially to participants over four weeks. All data was collected through self-report measures at prespecified intervals. Participants were instructed to complete a module or a set of two modules each week along with the required interval assessments. Email reminders alerted participants about the release of each module and the specified assessments. If the modules were not viewed in the first three days of their release, an automated email reminder was sent to participants and a second reminder was sent on the seventh day of their release. Similar automated email reminders at a similar frequency were also used for the self-report assessments. The personal dashboard was accessible to participants at any time. A “Contact Us” tab was available on the personal dashboard that allowed participants to contact the research team and ask questions about the study or express any concerns".

"The research team sent two follow-up emails, spaced two weeks apart, to participants who registered, were randomized, but had not begun treatment, serving as a reminder about the study and to gauge their ongoing interest in participating".

details about each module can be found in this table that has been included in the ms

Table 2. Sleep Best-i modules and treatment offered in each module.
Modules Offered Strategies
Module 1 (Psychoeducation) The module offered psychoeducation about sleep and insomnia, stages of sleep and neurobiology of sleep [14]. Offered at week 1.

Module 2-part 1 (Cognitive Restructuring and Sleep Hygiene) The module focused on the cognitive component of CBTi, types of unhelpful thoughts, methods to challenge unhelpful thoughts, and sleep hygiene [30]. Offered at week 1.

Module 2-part 2 (Sleep Scheduling and Stimulus Control) The module educated participants about specifying regular sleeping and waking up times with as little variation as possible between the two. Stimulus control restricted the bedroom or the bed to sleep only [34]. Offered at week 2.

Module 3 (Trauma, PTSD and Flashbacks) The module provided psychoeducation about trauma, PTSD, flashbacks and how trauma leads to sleep difficulties. It also provided behavioral interventions for trauma symptoms. Offered at week 3.

Module 4 (Nightmares) The module explored nightmare disorder, how nightmares develop, and how to rescript the nightmare into a more benign dream [16]. Offered at week 4.

Module 5, (Relapse Prevention) The module focused on identifying early warning signs and methods preventing relapse of symptoms [33]. Offered at week 4.

Mindfulness Module The module offered a recorded progressive muscle relaxation

mindfulness and it was available to participants throughout the study.

5-ix) Describe use parameters

Describe use parameters (e.g., intended “doses” and optimal timing for use). Clarify what instructions or recommendations were given to the user, e.g., regarding timing, frequency, heaviness of use, if any, or was the intervention used ad libitum.

	1	2	3	4	5	
subitem not at all important	○	◉	○	○	○	essential

Clear selection

Does your paper address subitem 5-ix?

Copy and paste relevant sections from the manuscript (include quotes in quotation marks "like this" to indicate direct quotes from your manuscript), or elaborate on this item by providing additional information not in the ms, or briefly explain why the item is not applicable/relevant for your study

Participants were encouraged to log in daily the site to complete sleep diary (not included in the manuscript). Each module was 17 minutes in duration, therefore, participants were encouraged to watch the entire module or the set of modules released for that week.

5-x) Clarify the level of human involvement

Clarify the level of human involvement (care providers or health professionals, also technical assistance) in the e-intervention or as co-intervention (detail number and expertise of professionals involved, if any, as well as “type of assistance offered, the timing and frequency of the support, how it is initiated, and the medium by which the assistance is delivered”. It may be necessary to distinguish between the level of human involvement required for the trial, and the level of human involvement required for a routine application outside of a RCT setting (discuss under item 21 – generalizability).

	1	2	3	4	5	
subitem not at all important	○	◉	○	○	○	essential

Clear selection

Does your paper address subitem 5-x?

Copy and paste relevant sections from the manuscript (include quotes in quotation marks "like this" to indicate direct quotes from your manuscript), or elaborate on this item by providing additional information not in the ms, or briefly explain why the item is not applicable/relevant for your study

The intervention was self-paced. Researchers of this study were involved only if participants asked questions through HealthZone.

5-xi) Report any prompts/reminders used

Report any prompts/reminders used: Clarify if there were prompts (letters, emails, phone calls, SMS) to use the application, what triggered them, frequency etc. It may be necessary to distinguish between the level of prompts/reminders required for the trial, and the level of prompts/reminders for a routine application outside of a RCT setting (discuss under item 21 – generalizability).

	1	2	3	4	5	
subitem not at all important	○	○	○	○	◉	essential

Clear selection

Does your paper address subitem 5-xi? *

Copy and paste relevant sections from the manuscript (include quotes in quotation marks "like this" to indicate direct quotes from your manuscript), or elaborate on this item by providing additional information not in the ms, or briefly explain why the item is not applicable/relevant for your study

"Automated email reminders alerted participants about the release of each module and the specified assessments. If the modules were not viewed in the first three days of their release, an automated email reminder was sent to participants and a second reminder was sent on the seventh day of their release. Similar automated email reminders at a similar frequency were also used for the self-report assessments".

5-xii) Describe any co-interventions (incl. training/support)

Describe any co-interventions (incl. training/support): Clearly state any interventions that are provided in addition to the targeted eHealth intervention, as ehealth intervention may not be designed as stand-alone intervention. This includes training sessions and support [1]. It may be necessary to distinguish between the level of training required for the trial, and the level of training for a routine application outside of a RCT setting (discuss under item 21 – generalizability.

	1	2	3	4	5	
subitem not at all important	(•)	( )	( )	( )	( )	essential

Clear selection

Does your paper address subitem 5-xii? *

Copy and paste relevant sections from the manuscript (include quotes in quotation marks "like this" to indicate direct quotes from your manuscript), or elaborate on this item by providing additional information not in the ms, or briefly explain why the item is not applicable/relevant for your study

Given the digital and self-paced nature of the program, no additional co-interventions, training, or support were provided to participants beyond the program's online content.

6a) Completely defined pre-specified primary and secondary outcome measures, including how and when they were assessed

Does your paper address CONSORT subitem 6a? *

Copy and paste relevant sections from the manuscript (include quotes in quotation marks "like this" to indicate direct quotes from your manuscript), or elaborate on this item by providing additional information not in the ms, or briefly explain why the item is not applicable/relevant for your study

"The treatment group completed self-report assessments at baseline, post-treatment following the application of Sleep Best-i and at 3-months follow-up. The waitlist group completed the same assessments at baseline, at pre-treatment (following 4 weeks of waiting and prior to crossing over to treatment), at post-treatment following the application of Sleep Best-i and at 3-months follow-up".

Primary outcome measures included: the Insomnia Severity Index (ISI), the Nightmare Disorder Index (NDI) and the PTSD CheckList – Civilian Version (PCL-5).

Secondary outcome measures included: Generalized Anxiety Disorder Questionnaire (GAD-7), Patient Health Questionnaire (PHQ-9), and the Pittsburgh Sleep Quality Index (PSQI),

6a-i) Online questionnaires: describe if they were validated for online use and apply CHERRIES items to describe how the questionnaires were designed/deployed

If outcomes were obtained through online questionnaires, describe if they were validated for online use and apply CHERRIES items to describe how the questionnaires were designed/deployed [9].

	1	2	3	4	5	
subitem not at all important	○	○	○	◉	○	essential

Clear selection

Does your paper address subitem 6a-i?

Copy and paste relevant sections from manuscript text

"The self-report measures as well as the modules were tested by two research team members (FI and BK) before being released to participants".

6a-ii) Describe whether and how “use” (including intensity of use/dosage) was defined/measured/monitored

Describe whether and how “use” (including intensity of use/dosage) was defined/measured/monitored (logins, logfile analysis, etc.). Use/adoption metrics are important process outcomes that should be reported in any ehealth trial.

	1	2	3	4	5	
subitem not at all important	○	○	○	◉	○	essential

Clear selection

Does your paper address subitem 6a-ii?

Copy and paste relevant sections from manuscript text

"To assess level of engagement with treatment the number of logins and duration of time each participant spent on the site was monitored".

"HelalthZone recorded data about the number of logins, date and duration for each participant. Participants were expected to spend at least three hours over the four weeks to indicate adherence to treatment. This time is optimal for viewing all modules and completing all assessments".

6a-iii) Describe whether, how, and when qualitative feedback from participants was obtained

Describe whether, how, and when qualitative feedback from participants was obtained (e.g., through emails, feedback forms, interviews, focus groups).

	1	2	3	4	5	
subitem not at all important	○	◉	○	○	○	essential

Clear selection

Does your paper address subitem 6a-iii?

Copy and paste relevant sections from manuscript text

Although not reported in the present manuscript, feedback on the modules was collected from participants at weeks 2, 3, 4, and 5 through brief descriptive evaluations.

6b) Any changes to trial outcomes after the trial commenced, with reasons

Does your paper address CONSORT subitem 6b? *

Copy and paste relevant sections from the manuscript (include quotes in quotation marks "like this" to indicate direct quotes from your manuscript), or elaborate on this item by providing additional information not in the ms, or briefly explain why the item is not applicable/relevant for your study

"Since its initiation, the protocol has undergone only minor revisions, specifically an update to the statistical analysis methodology to include linear mixed methods and ITT analysis. The desired sample size was not achieved due to the project's timeline, resulting in a smaller sample size than anticipated".

7a) How sample size was determined

NPT: When applicable, details of whether and how the clustering by care provides or centers was addressed

7a-i) Describe whether and how expected attrition was taken into account when calculating the sample size

Describe whether and how expected attrition was taken into account when calculating the sample size.

	1	2	3	4	5	
subitem not at all important	○	○	○	○	◉	essential

Clear selection

Does your paper address subitem 7a-i?

Copy and paste relevant sections from manuscript title (include quotes in quotation marks "like this" to indicate direct quotes from your manuscript), or elaborate on this item by providing additional information not in the ms, or briefly explain why the item is not applicable/relevant for your study

"A priori power analysis indicated a required sample size of n=50 to detect meaningful differences between the intervention and waitlist groups, considering α=.05, d= 1.0, 20% attrition, and 80% power [29]".

7b) When applicable, explanation of any interim analyses and stopping guidelines

Does your paper address CONSORT subitem 7b? *

Copy and paste relevant sections from the manuscript (include quotes in quotation marks "like this" to indicate direct quotes from your manuscript), or elaborate on this item by providing additional information not in the ms, or briefly explain why the item is not applicable/relevant for your study

"No statistical analyses were performed in the interim of the study".

8a) Method used to generate the random allocation sequence
NPT: When applicable, how care providers were allocated to each trial group

Does your paper address CONSORT subitem 8a? *

Copy and paste relevant sections from the manuscript (include quotes in quotation marks "like this" to indicate direct quotes from your manuscript), or elaborate on this item by providing additional information not in the ms, or briefly explain why the item is not applicable/relevant for your study

"This study used a parallel arm, sequential alternation method of assigning participants to either intervention or waitlist groups in a 1:1 ratio".

8b) Type of randomisation; details of any restriction (such as blocking and block size)

Does your paper address CONSORT subitem 8b? *

Copy and paste relevant sections from the manuscript (include quotes in quotation marks "like this" to indicate direct quotes from your manuscript), or elaborate on this item by providing additional information not in the ms, or briefly explain why the item is not applicable/relevant for your study

"Participants were sequentially allocated via simple randomization in the order of their enrolment, using a computer-generated simple randomization sequence".

9) Mechanism used to implement the random allocation sequence (such as sequentially numbered containers), describing any steps taken to conceal the sequence until interventions were assigned

Does your paper address CONSORT subitem 9? *

Copy and paste relevant sections from the manuscript (include quotes in quotation marks "like this" to indicate direct quotes from your manuscript), or elaborate on this item by providing additional information not in the ms, or briefly explain why the item is not applicable/relevant for your study

"Participants were sequentially allocated via simple randomization in the order of their enrolment, using a computer-generated simple randomization sequence".

10) Who generated the random allocation sequence, who enrolled participants, and who assigned participants to interventions

Does your paper address CONSORT subitem 10? *

Copy and paste relevant sections from the manuscript (include quotes in quotation marks "like this" to indicate direct quotes from your manuscript), or elaborate on this item by providing additional information not in the ms, or briefly explain why the item is not applicable/relevant for your study

"The baseline data were viewed by two researchers to decide eligibility. Those who were eligible (assessed by FI and GK, both clinical psychologists) were contacted via email to notify them of their eligibility, their randomization and giving them instructions on how to access their personal dashboard on HealthZone".

11a) If done, who was blinded after assignment to interventions (for example, participants, care providers, those assessing outcomes) and how

NPT: Whether or not administering co-interventions were blinded to group assignment

11a-i) Specify who was blinded, and who wasn't

Specify who was blinded, and who wasn't. Usually, in web-based trials it is not possible to blind the participants [1, 3] (this should be clearly acknowledged), but it may be possible to blind outcome assessors, those doing data analysis or those administering co-interventions (if any).

	1	2	3	4	5	
subitem not at all important	○	○	○	○	◉	essential

Clear selection

Does your paper address subitem 11a-i? *

Copy and paste relevant sections from the manuscript (include quotes in quotation marks "like this" to indicate direct quotes from your manuscript), or elaborate on this item by providing additional information not in the ms, or briefly explain why the item is not applicable/relevant for your study

"Participants were informed about the purpose, allocation, and the structure of the study as explained in the PLIS. However, they were not informed about the study's hypotheses".

Researchers were not blinded.

11a-ii) Discuss e.g., whether participants knew which intervention was the “intervention of interest” and which one was the “comparator”

Informed consent procedures (4a-ii) can create biases and certain expectations - discuss e.g., whether participants knew which intervention was the “intervention of interest” and which one was the “comparator”.

	1	2	3	4	5	
subitem not at all important	○	○	○	◉	○	essential

Clear selection

Does your paper address subitem 11a-ii?

Copy and paste relevant sections from the manuscript (include quotes in quotation marks "like this" to indicate direct quotes from your manuscript), or elaborate on this item by providing additional information not in the ms, or briefly explain why the item is not applicable/relevant for your study

Participants knew if they were assigned to the intervention or the waitlist group. However, they were not aware of the study's hypotheses.

11b) If relevant, description of the similarity of interventions

(this item is usually not relevant for ehealth trials as it refers to similarity of a placebo or sham intervention to a active medication/intervention)

Does your paper address CONSORT subitem 11b? *

Copy and paste relevant sections from the manuscript (include quotes in quotation marks "like this" to indicate direct quotes from your manuscript), or elaborate on this item by providing additional information not in the ms, or briefly explain why the item is not applicable/relevant for your study

The waitlist group received the same treatment as the intervention group.

12a) Statistical methods used to compare groups for primary and secondary outcomes

NPT: When applicable, details of whether and how the clustering by care providers or centers was addressed

Does your paper address CONSORT subitem 12a? *

Copy and paste relevant sections from the manuscript (include quotes in quotation marks "like this" to indicate direct quotes from your manuscript), or elaborate on this item by providing additional information not in the ms, or briefly explain why the item is not applicable/relevant for your study

"To ensure a robust evaluation of the intervention's feasibility, the Intention-To-Treat (ITT) with n=30, as well as Per Protocol (PP) analysis with a sample of n=20 were utilized allowing for a comprehensive assessment of treatment outcomes, thereby enhancing the accuracy and generalizability of the findings [28,50,51]".

12a-i) Imputation techniques to deal with attrition / missing values

Imputation techniques to deal with attrition / missing values: Not all participants will use the intervention/comparator as intended and attrition is typically high in ehealth trials. Specify how participants who did not use the application or dropped out from the trial were treated in the statistical analysis (a complete case analysis is strongly discouraged, and simple imputation techniques such as LOCF may also be problematic [4]).

	1	2	3	4	5	
subitem not at all important	○	○	○	○	●	essential

Clear selection

Does your paper address subitem 12a-i? *

Copy and paste relevant sections from the manuscript (include quotes in quotation marks "like this" to indicate direct quotes from your manuscript), or elaborate on this item by providing additional information not in the ms, or briefly explain why the item is not applicable/relevant for your study

"For both analyses, missing data was addressed using multiple imputation by chained equations (MICE). This approach is more robust than other methods, such as single imputation, because it generates multiple predictions for each missing value, accounting for uncertainty in the imputations and yielding more accurate standard errors [52]".

"To address uncertainty, each missing value was imputed twenty times, using predictive mean matching based on the three nearest values, across multiple iterations to create 100 imputed datasets. The imputed values were then averaged to create the final estimates [53]. Predictive mean matching, a partially parametric method, is preferable to fully parametric linear regression, as it remains effective even when the normality assumption of the underlying variable is violated. This method also helps preserve the distribution of observed values in the missing data [54]".

12b) Methods for additional analyses, such as subgroup analyses and adjusted analyses

Does your paper address CONSORT subitem 12b? *

Copy and paste relevant sections from the manuscript (include quotes in quotation marks "like this" to indicate direct quotes from your manuscript), or elaborate on this item by providing additional information not in the ms, or briefly explain why the item is not applicable/relevant for your study

"To address the third hypothesis, separate mixed-effects linear models' analyses were performed for each group (intervention and waitlist) on all primary and secondary outcome measures. The analysis incorporated data from the following time points: baseline, post-treatment, and 3-months follow-up for the intervention group, and pre-treatment, post-treatment, and 3-months follow-up for the waitlist group".

X26) REB/IRB Approval and Ethical Considerations [recommended as subheading under "Methods"] (not a CONSORT item)

X26-i) Comment on ethics committee approval

	1	2	3	4	5	
subitem not at all important	○	○	○	○	◉	essential

Clear selection

Does your paper address subitem X26-i?

Copy and paste relevant sections from the manuscript (include quotes in quotation marks "like this" to indicate direct quotes from your manuscript), or elaborate on this item by providing additional information not in the ms, or briefly explain why the item is not applicable/relevant for your study

"Ethical Considerations
Following the ethics approval from Federation University human research ethics committee/Australia (Approval #: 2022-153), an advertisement about the study was distributed. Wildfire survivors interested in the study read a Plain Language Information Statement (PLIS) about the study and provided baseline data. Participants who met the selection criteria provided informed consent by checking a box on a digital consent form hosted on Federation University's HealthZone platform, thereby indicating their agreement to participate in the study. Participants were informed that their participation was voluntary. To ensure the secure storage and handling of collected data, it was stored on a password-protected platform (HealthZone) accessible only to the researchers involved in the study. To protect participant confidentiality, names were replaced with unique identifiers, and only country of residence was collected, with no addresses recorded. The data will be retained for a period of five years before being destroyed. To manage any potential risks, participants were provided with emergency contact numbers relevant to their country of residence".

x26-ii) Outline informed consent procedures

Outline informed consent procedures e.g., if consent was obtained offline or online (how? Checkbox, etc.?), and what information was provided (see 4a-ii). See [6] for some items to be included in informed consent documents.

	1	2	3	4	5	
subitem not at all important	○	○	○	○	◉	essential

Clear selection

Does your paper address subitem X26-ii?

Copy and paste relevant sections from the manuscript (include quotes in quotation marks "like this" to indicate direct quotes from your manuscript), or elaborate on this item by providing additional information not in the ms, or briefly explain why the item is not applicable/relevant for your study

"Participants who met the selection criteria provided informed consent by checking a box on a digital consent form hosted on Federation University's HealthZone platform, thereby indicating their agreement to participate in the study".

X26-iii) Safety and security procedures

Safety and security procedures, incl. privacy considerations, and any steps taken to reduce the likelihood or detection of harm (e.g., education and training, availability of a hotline)

	1	2	3	4	5	
subitem not at all important	○	○	○	○	◉	essential

Clear selection

Does your paper address subitem X26-iii?

Copy and paste relevant sections from the manuscript (include quotes in quotation marks "like this" to indicate direct quotes from your manuscript), or elaborate on this item by providing additional information not in the ms, or briefly explain why the item is not applicable/relevant for your study

"To ensure the secure storage and handling of collected data, it was stored on a password-protected platform (HealthZone) accessible only to the researchers involved in the study. To protect participant confidentiality, names were replaced with unique identifiers, and only country of residence was collected, with no addresses recorded. The data will be retained for a period of five years before being destroyed".

RESULTS

13a) For each group, the numbers of participants who were randomly assigned, received intended treatment, and were analysed for the primary outcome

NPT: The number of care providers or centers performing the intervention in each group and the number of patients treated by each care provider in each center

Does your paper address CONSORT subitem 13a? *

Copy and paste relevant sections from the manuscript (include quotes in quotation marks "like this" to indicate direct quotes from your manuscript), or elaborate on this item by providing additional information not in the ms, or briefly explain why the item is not applicable/relevant for your study

"Thirty-three participants responded to the advertisement, registered an account with Federation University's digital platform/ HealthZone, and provided baseline data. Three were excluded and 30 were randomised to either the intervention or the waitlist groups. Sixteen (53.33%) were randomized into the intervention group and fourteen (46.67%) were in the waitlist group (for the participants' CONSORT chart refer to Figure 1). Age ranged between 18 and 79 years (M = 52.5, SD = 16.26, N=30), with the majority being females 19 (63.3%), vs 11(36.7%) males. Twenty participants completed the trial, and their data were analyzed as per protocol. Their ages ranged from 18 to 79 years (M = 53.75, SD = 16.54) and most were females 14 (70%), vs 6 (30%) males. Demographic variables for the treatment and waitlist groups are shown in Table 1".

13b) For each group, losses and exclusions after randomisation, together with reasons

Does your paper address CONSORT subitem 13b? (NOTE: Preferably, this is shown in a CONSORT flow diagram) *

Copy and paste relevant sections from the manuscript (include quotes in quotation marks "like this" to indicate direct quotes from your manuscript), or elaborate on this item by providing additional information not in the ms, or briefly explain why the item is not applicable/relevant for your study

"Four participants were excluded from the PP analysis for the following reasons: two participants, who completed the pilot trial and provided complete set of data revealed they had restless leg syndrome that was not initially reported during the screening process; one participant received one treatment module, however, withdrew from the study due to illness providing only baseline measures; and a fourth participant who provided and completed 50% of data and modules withdrew due to illness and family commitments. Therefore, the PP analysis was conducted on a sample of n=20. Six participants provided baseline data, however, did not initiate treatment and did not respond to emails sent by the research team. Table 1 indicates that there were no significant differences in demographic variables between the intervention and waitlist groups at baseline assessment (all P>.05)".

13b-i) Attrition diagram

Strongly recommended: An attrition diagram (e.g., proportion of participants still logging in or using the intervention/comparator in each group plotted over time, similar to a survival curve) or other figures or tables demonstrating usage/dose/engagement.

	1	2	3	4	5	
subitem not at all important	○	○	◉	○	○	essential

Clear selection

Does your paper address subitem 13b-i?

Copy and paste relevant sections from the manuscript or cite the figure number if applicable (include quotes in quotation marks "like this" to indicate direct quotes from your manuscript), or elaborate on this item by providing additional information not in the ms, or briefly explain why the item is not applicable/relevant for your study

Given the pilot nature of this study, participant attrition rates and platform usage were monitored and documented within HealthZone. However, due to the small sample size (n = 30), the number of participants who dropped out at each stage was too small to warrant a detailed attrition diagram, as it would not provide meaningful insights into the patterns of attrition. Additionally, the limited sample size would make it difficult to maintain participant anonymity, potentially compromising confidentiality. Therefore, a brief description of attrition rates and reasons for withdrawal was provided instead.

14a) Dates defining the periods of recruitment and follow-up

Does your paper address CONSORT subitem 14a? *

Copy and paste relevant sections from the manuscript (include quotes in quotation marks "like this" to indicate direct quotes from your manuscript), or elaborate on this item by providing additional information not in the ms, or briefly explain why the item is not applicable/relevant for your study

"Participants were recruited between May 2023 and December 2023 via social media platforms including international Facebook campaigns, radio broadcasts, LinkedIn, Reddit, and online community noticeboards".

"The treatment group completed self-report assessments at baseline, post-treatment following the application of Sleep Best-i and at 3-months follow-up. The waitlist group completed the same assessments at baseline, at pre-treatment (following 4 weeks of waiting and prior to crossing over to treatment), at post-treatment following the application of Sleep Best-i and at 3-months follow-up".

14a-i) Indicate if critical “secular events” fell into the study period

Indicate if critical “secular events” fell into the study period, e.g., significant changes in Internet resources available or “changes in computer hardware or Internet delivery resources”

	1	2	3	4	5	
subitem not at all important	○	○	○	◉	○	essential

Clear selection

Does your paper address subitem 14a-i?

Copy and paste relevant sections from the manuscript (include quotes in quotation marks "like this" to indicate direct quotes from your manuscript), or elaborate on this item by providing additional information not in the ms, or briefly explain why the item is not applicable/relevant for your study

No significant external events or major secular trends that could potentially impact the study outcomes occurred during the trial period.

14b) Why the trial ended or was stopped (early)

Does your paper address CONSORT subitem 14b? *

Copy and paste relevant sections from the manuscript (include quotes in quotation marks "like this" to indicate direct quotes from your manuscript), or elaborate on this item by providing additional information not in the ms, or briefly explain why the item is not applicable/relevant for your study

The project's timeline elapsed, necessitating the premature termination of the trial before achieving the target sample size.

15) A table showing baseline demographic and clinical characteristics for each group

NPT: When applicable, a description of care providers (case volume, qualification, expertise, etc.) and centers (volume) in each group

Does your paper address CONSORT subitem 15? *

Copy and paste relevant sections from the manuscript (include quotes in quotation marks "like this" to indicate direct quotes from your manuscript), or elaborate on this item by providing additional information not in the ms, or briefly explain why the item is not applicable/relevant for your study

Table 1 in the manuscript addresses subitem 15

15-i) Report demographics associated with digital divide issues

In ehealth trials it is particularly important to report demographics associated with digital divide issues, such as age, education, gender, social-economic status, computer/Internet/ehealth literacy of the participants, if known.

	1	2	3	4	5	
subitem not at all important	○	○	○	○	◉	essential

Clear selection

Does your paper address subitem 15-i? *

Copy and paste relevant sections from the manuscript (include quotes in quotation marks "like this" to indicate direct quotes from your manuscript), or elaborate on this item by providing additional information not in the ms, or briefly explain why the item is not applicable/relevant for your study

Table 1 in the manuscript provides information related to subitem 15-i

16) For each group, number of participants (denominator) included in each analysis and whether the analysis was by original assigned groups

16-i) Report multiple “denominators” and provide definitions

Report multiple “denominators” and provide definitions: Report N’s (and effect sizes) “across a range of study participation [and use] thresholds” [1], e.g., N exposed, N consented, N used more than x times, N used more than y weeks, N participants “used” the intervention/comparator at specific pre-defined time points of interest (in absolute and relative numbers per group). Always clearly define “use” of the intervention.

	1	2	3	4	5	
subitem not at all important	○	○	○	○	●	essential

Clear selection

Does your paper address subitem 16-i? *

Copy and paste relevant sections from the manuscript (include quotes in quotation marks "like this" to indicate direct quotes from your manuscript), or elaborate on this item by providing additional information not in the ms, or briefly explain why the item is not applicable/relevant for your study

"To ensure a robust evaluation of the intervention's feasibility, the Intention-To-Treat (ITT) with n=30, as well as Per Protocol (PP) analysis with a sample of n=20 were utilized allowing for a comprehensive assessment of treatment outcomes, thereby enhancing the accuracy and generalizability of the findings [28,50,51]".

"Adjusted analyses of intervention effects were conducted using mixed-effects linear regression models. Both fixed and random effects were estimated to assess the impact of the intervention on the change in all outcomes (primary and secondary) over time and by condition. Given that observed covariates were balanced between the two conditions (waitlist and treatment), the difference-in-difference (DID) effect for the fixed part of the mixed-effects models was tested by including interaction terms for time point and condition. DID is considered the most appropriate measure to assess causal effects in time series design as it effectively controls for time-invariant confounding variables. By doing so, DID reduces bias and enhances internal validity, providing a more accurate estimate of the treatment effect [56]".

Due to the limited sample size, only basic usage metrics were tracked, specifically the number of visits, whereas more detailed analyses and outcomes were not feasible.

16-ii) Primary analysis should be intent-to-treat

Primary analysis should be intent-to-treat, secondary analyses could include comparing only “users”, with the appropriate caveats that this is no longer a randomized sample (see 18-i).

	1	2	3	4	5	
subitem not at all important	○	○	○	○	◉	essential

Clear selection

Does your paper address subitem 16-ii?

Copy and paste relevant sections from the manuscript (include quotes in quotation marks "like this" to indicate direct quotes from your manuscript), or elaborate on this item by providing additional information not in the ms, or briefly explain why the item is not applicable/relevant for your study

Yes, ITT was used as the principal analysis

17a) For each primary and secondary outcome, results for each group, and the estimated effect size and its precision (such as 95% confidence interval)

Does your paper address CONSORT subitem 17a? *

Copy and paste relevant sections from the manuscript (include quotes in quotation marks "like this" to indicate direct quotes from your manuscript), or elaborate on this item by providing additional information not in the ms, or briefly explain why the item is not applicable/relevant for your study

Yes, all the information is provided in tables 3-14.

17a-i) Presentation of process outcomes such as metrics of use and intensity of use

In addition to primary/secondary (clinical) outcomes, the presentation of process outcomes such as metrics of use and intensity of use (dose, exposure) and their operational definitions is critical. This does not only refer to metrics of attrition (13-b) (often a binary variable), but also to more continuous exposure metrics such as “average session length”. These must be accompanied by a technical description how a metric like a “session” is defined (e.g., timeout after idle time) [1] (report under item 6a).

	1	2	3	4	5	
subitem not at all important	○	○	○	○	◉	essential

Clear selection

Does your paper address subitem 17a-i?

Copy and paste relevant sections from the manuscript (include quotes in quotation marks "like this" to indicate direct quotes from your manuscript), or elaborate on this item by providing additional information not in the ms, or briefly explain why the item is not applicable/relevant for your study

"The number of logins into the site was observed as an indicator of the level of engagement by participants with the modules and it was found that the 20 participants who were included in the PP analysis had an average of M = 8.5 logins times during their engagement with Sleep Best-i. Approximately 5% logged in 12 times to the site, 5% logged in 5 times, 5% logged in 7 times, 10% logged in 6 times, 15% logged in 11 times, 20% logged in 8, 9, and 10 times respectively. On average, participants spent 116 minutes onsite visiting modules and completing assessments".

17b) For binary outcomes, presentation of both absolute and relative effect sizes is recommended

Does your paper address CONSORT subitem 17b? *

Copy and paste relevant sections from the manuscript (include quotes in quotation marks "like this" to indicate direct quotes from your manuscript), or elaborate on this item by providing additional information not in the ms, or briefly explain why the item is not applicable/relevant for your study

We employed a Difference-in-Differences (DID) analysis to estimate the treatment effects, quantifying the magnitude of change in both primary and secondary outcomes. This approach allowed us to measure the power of the intervention by comparing the pre-post changes in the treatment group to those in the control group.

18) Results of any other analyses performed, including subgroup analyses and adjusted analyses, distinguishing pre-specified from exploratory

Does your paper address CONSORT subitem 18? *

Copy and paste relevant sections from the manuscript (include quotes in quotation marks "like this" to indicate direct quotes from your manuscript), or elaborate on this item by providing additional information not in the ms, or briefly explain why the item is not applicable/relevant for your study

We included a clinical significance analysis in the manuscript.

"Clinical Significance

Figure 8 shows the number of participants who experienced clinical significance using the ITT analysis from baseline to 3-months follow-up assessments. Following receiving Sleep Best-i, a higher percentage of participants from the waitlist group experienced clinical significance of their symptoms of nightmares and PTSD than the treatment group, however, this difference between the two groups was not significant (Table 15). Two participants did not provide a 3-months follow-up data on the ISI, NDI and the PLC-5. Table 16 shows comparable results to those reported in the ITT analysis with higher percentage of participants from the waitlist group experiencing clinical significance on the NDI and the PCL-5 than the treatment group".

18-i) Subgroup analysis of comparing only users

A subgroup analysis of comparing only users is not uncommon in ehealth trials, but if done, it must be stressed that this is a self-selected sample and no longer an unbiased sample from a randomized trial (see 16-iii).

	1	2	3	4	5	
subitem not at all important	○	○	○	○	◉	essential

Clear selection

Does your paper address subitem 18-i?

Copy and paste relevant sections from the manuscript (include quotes in quotation marks "like this" to indicate direct quotes from your manuscript), or elaborate on this item by providing additional information not in the ms, or briefly explain why the item is not applicable/relevant for your study

Yes, we included PP analysis as a secondary analysis.

19) All important harms or unintended effects in each group
(for specific guidance see CONSORT for harms)

Does your paper address CONSORT subitem 19? *

Copy and paste relevant sections from the manuscript (include quotes in quotation marks "like this" to indicate direct quotes from your manuscript), or elaborate on this item by providing additional information not in the ms, or briefly explain why the item is not applicable/relevant for your study

Harm was not reported by participation in this trial.

19-i) Include privacy breaches, technical problems

Include privacy breaches, technical problems. This does not only include physical "harm" to participants, but also incidents such as perceived or real privacy breaches [1], technical problems, and other unexpected/unintended incidents. "Unintended effects" also includes unintended positive effects [2].

	1	2	3	4	5	
subitem not at all important	○	○	○	◉	○	essential

Clear selection

Does your paper address subitem 19-i?

Copy and paste relevant sections from the manuscript (include quotes in quotation marks "like this" to indicate direct quotes from your manuscript), or elaborate on this item by providing additional information not in the ms, or briefly explain why the item is not applicable/relevant for your study

The study was free from any significant technical issues or glitches that would warrant mention, and no privacy breaches or concerns were reported to the research team throughout the duration of the project.

19-ii) Include qualitative feedback from participants or observations from staff/researchers

Include qualitative feedback from participants or observations from staff/researchers, if available, on strengths and shortcomings of the application, especially if they point to unintended/unexpected effects or uses. This includes (if available) reasons for why people did or did not use the application as intended by the developers.

	1	2	3	4	5	
subitem not at all important	○	○	◉	○	○	essential

Clear selection

Does your paper address subitem 19-ii?

Copy and paste relevant sections from the manuscript (include quotes in quotation marks "like this" to indicate direct quotes from your manuscript), or elaborate on this item by providing additional information not in the ms, or briefly explain why the item is not applicable/relevant for your study

Feedback was collected from participants throughout the project, but it is not included in this manuscript. A separate analysis of this feedback is planned, and the results will be presented in a subsequent publication, offering additional insights into the participants' responses and suggestions.

DISCUSSION

22) Interpretation consistent with results, balancing benefits and harms, and considering other relevant evidence

NPT: In addition, take into account the choice of the comparator, lack of or partial blinding, and unequal expertise of care providers or centers in each group

22-i) Restate study questions and summarize the answers suggested by the data, starting with primary outcomes and process outcomes (use)

Restate study questions and summarize the answers suggested by the data, starting with primary outcomes and process outcomes (use).

	1	2	3	4	5	
subitem not at all important	○	○	○	○	◉	essential

Clear selection

Does your paper address subitem 22-i? *

Copy and paste relevant sections from the manuscript (include quotes in quotation marks "like this" to indicate direct quotes from your manuscript), or elaborate on this item by providing additional information not in the ms, or briefly explain why the item is not applicable/relevant for your study

"The aim of this clinical pilot trial was to assess the feasibility of a brief (6 modules over four weeks), self-paced, digital intervention for the treatment of insomnia, nightmares and PTSD symptoms in an international sample of wildfire survivors. Our first hypothesis received partial support from the PP analysis, which revealed a significant interaction effect of condition x time. This indicates that Sleep Best-i effectively reduced symptoms of nightmares and PTSD from pre- to post-intervention in the treatment group, compared to the waitlist group. However, no significant changes were observed in insomnia symptoms. The ITT analysis yielded similar findings, with a significant main effect of time showing a reduction in nightmare and PTSD symptoms at post-treatment for the intervention group, but no significant changes in insomnia symptoms. In examining the two groups separately, Sleep Best-i significantly reduced symptoms of insomnia, nightmares and PTSD from baseline to post-intervention and this improvement in symptoms was maintained at 3-months assessment for the two groups across both the PP and ITT analyses".

"In relation to the second hypothesis, the PP analysis revealed that Sleep Best-i not only alleviated symptoms of nightmares and trauma but also significantly reduced anxiety symptoms (main effect of condition) in the treatment group compared to the waitlist group at post-intervention. Additionally, the treatment group experienced a significant reduction in depressive symptoms at post-treatment (main effect of time) and a significant interaction effect of time x condition on sleep quality, indicating more pronounced improvements in sleep quality at post-intervention compared to the waitlist group. The ITT analysis yielded similar findings, with significant reductions in depressive symptoms at post-intervention and a significant interaction effect of time x condition on sleep quality. However, the reduction in anxiety symptoms was no longer significant in the ITT analysis. This is notable, as research suggests a strong relationship between insomnia and anxiety symptoms, with the expectation that successful insomnia treatment would lead to a reduction in anxiety symptoms [65]".

22-ii) Highlight unanswered new questions, suggest future research

Highlight unanswered new questions, suggest future research.

	1	2	3	4	5	
subitem not at all important	○	○	○	○	◉	essential

Clear selection

Does your paper address subitem 22-ii?

Copy and paste relevant sections from the manuscript (include quotes in quotation marks "like this" to indicate direct quotes from your manuscript), or elaborate on this item by providing additional information not in the ms, or briefly explain why the item is not applicable/relevant for your study

"The clinical pilot trial has limitations that warrant consideration. The sample consisted of self-selected individuals, and the absence of clinical assessments to confirm diagnoses of insomnia, nightmares, and PTSD disorders introduces a potential source of bias. The small sample size is another impediment, underscoring the need for further testing with a larger and more diverse population to establish the external validity of the intervention".

20) Trial limitations, addressing sources of potential bias, imprecision, and, if relevant, multiplicity of analyses

20-i) Typical limitations in ehealth trials

Typical limitations in ehealth trials: Participants in ehealth trials are rarely blinded. Ehealth trials often look at a multiplicity of outcomes, increasing risk for a Type I error. Discuss biases due to non-use of the intervention/usability issues, biases through informed consent procedures, unexpected events.

	1	2	3	4	5	
subitem not at all important	○	○	○	○	●	essential

Clear selection

Does your paper address subitem 20-i? *

Copy and paste relevant sections from the manuscript (include quotes in quotation marks "like this" to indicate direct quotes from your manuscript), or elaborate on this item by providing additional information not in the ms, or briefly explain why the item is not applicable/relevant for your study

"Limitations
The clinical pilot trial has limitations that warrant consideration. The sample consisted of self-selected individuals, and the absence of clinical assessments to confirm diagnoses of insomnia, nightmares, and PTSD disorders introduces a potential source of bias. The small sample size is another impediment, underscoring the need for further testing with a larger and more diverse population to establish the external validity of the intervention. One major concern in relation to Sleep Best-i is the PTSD module and the potential for triggering trauma symptoms. Although this risk was anticipated, participants were provided with emergency numbers. Additionally, during the initial two weeks of treatment, participants were provided with cognitive and behavioral techniques to manage stress. Our study was also an open label trial. future research should address the aforementioned gaps".

21) Generalisability (external validity, applicability) of the trial findings

NPT: External validity of the trial findings according to the intervention, comparators, patients, and care providers or centers involved in the trial

21-i) Generalizability to other populations

Generalizability to other populations: In particular, discuss generalizability to a general Internet population, outside of a RCT setting, and general patient population, including applicability of the study results for other organizations

	1	2	3	4	5	
subitem not at all important	○	○	○	○	◉	essential

Clear selection

Does your paper address subitem 21-i?

Copy and paste relevant sections from the manuscript (include quotes in quotation marks "like this" to indicate direct quotes from your manuscript), or elaborate on this item by providing additional information not in the ms, or briefly explain why the item is not applicable/relevant for your study

"The small sample size is another impediment, underscoring the need for further testing with a larger and more diverse population to establish the external validity of the intervention".

21-ii) Discuss if there were elements in the RCT that would be different in a routine application setting

Discuss if there were elements in the RCT that would be different in a routine application setting (e.g., prompts/reminders, more human involvement, training sessions or other co-interventions) and what impact the omission of these elements could have on use, adoption, or outcomes if the intervention is applied outside of a RCT setting.

	1	2	3	4	5	
subitem not at all important	○	◉	○	○	○	essential

Clear selection

Does your paper address subitem 21-ii?

Copy and paste relevant sections from the manuscript (include quotes in quotation marks "like this" to indicate direct quotes from your manuscript), or elaborate on this item by providing additional information not in the ms, or briefly explain why the item is not applicable/relevant for your study

This is not applicable for our study.

OTHER INFORMATION

23) Registration number and name of trial registry

Does your paper address CONSORT subitem 23? *

Copy and paste relevant sections from the manuscript (include quotes in quotation marks "like this" to indicate direct quotes from your manuscript), or elaborate on this item by providing additional information not in the ms, or briefly explain why the item is not applicable/relevant for your study

"The trial was registered in the Australian New Zealand Clinical Trial Registry (Trial ID: ACTRN12623000415606)".

"Clinical trial name: Sleep Best-i: An online cognitive-behavioral intervention for the treatment of insomnia and nightmares in wildfire survivors
Trial ID: ACTRN12623000415606
URL: https://anzctr.org.au/Trial/Registration/TrialReview.aspx?id=385054
Registry: Australian New Zealand Clinical Trial Registry"

24) Where the full trial protocol can be accessed, if available

Does your paper address CONSORT subitem 24? *

Cite a Multimedia Appendix, other reference, or copy and paste relevant sections from the manuscript (include quotes in quotation marks "like this" to indicate direct quotes from your manuscript), or elaborate on this item by providing additional information not in the ms, or briefly explain why the item is not applicable/relevant for your study

A CONSORT diagram is provided in the manuscript. Data and full protocol of the study can be requested from the corresponding author.

25) Sources of funding and other support (such as supply of drugs), role of funders

Does your paper address CONSORT subitem 25? *

Copy and paste relevant sections from the manuscript (include quotes in quotation marks "like this" to indicate direct quotes from your manuscript), or elaborate on this item by providing additional information not in the ms, or briefly explain why the item is not applicable/relevant for your study

Funding Statement: this study received funding from the Natural Hazards Research Australia through an awarded scholarship to FI.

X27) Conflicts of Interest (not a CONSORT item)

X27-i) State the relation of the study team towards the system being evaluated

In addition to the usual declaration of interests (financial or otherwise), also state the relation of the study team towards the system being evaluated, i.e., state if the authors/evaluators are distinct from or identical with the developers/sponsors of the intervention.

	1	2	3	4	5	
subitem not at all important	○	○	○	○	◉	essential

Clear selection

Does your paper address subitem X27-i?

Copy and paste relevant sections from the manuscript (include quotes in quotation marks "like this" to indicate direct quotes from your manuscript), or elaborate on this item by providing additional information not in the ms, or briefly explain why the item is not applicable/relevant for your study

The researchers who designed and developed the Sleep Best-i program also conducted this study, serving as both the intervention's creators and the trial's implementers.

About the CONSORT EHEALTH checklist

As a result of using this checklist, did you make changes in your manuscript? *

- ◉ yes, major changes
- ○ yes, minor changes
- ○ no

What were the most important changes you made as a result of using this checklist?

We revised and added a wealth of information to the methods and results sections.

How much time did you spend on going through the checklist INCLUDING making changes in your manuscript *

There has been a lot of work done, that was probably equal to 2 weeks of work on the manuscript and the checklist.

As a result of using this checklist, do you think your manuscript has improved? *

- (selected) yes
- no
- Other:

Would you like to become involved in the CONSORT EHEALTH group?

This would involve for example becoming involved in participating in a workshop and writing an "Explanation and Elaboration" document

- yes
- (selected) no
- Other:

Clear selection

Any other comments or questions on CONSORT EHEALTH

Your answer

STOP - Save this form as PDF before you click submit

To generate a record that you filled in this form, we recommend to generate a PDF of this page (on a Mac, simply select "print" and then select "print as PDF") before you submit it.

When you submit your (revised) paper to JMIR, please upload the PDF as supplementary file.

Don't worry if some text in the textboxes is cut off, as we still have the complete information in our database. Thank you!

Final step: Click submit !

Click submit so we have your answers in our database!

Submit Clear form

Never submit passwords through Google Forms.

This content is neither created nor endorsed by Google. Report Abuse - Terms of Service - Privacy Policy

Google Forms
