# Supplementary material for: Digital Cognitive Behavioral Therapy–Based Treatment for Insomnia, Nightmares, and Posttraumatic Stress Disorder Symptoms in Survivors of Wildfires: Pilot Randomized Feasibility Trial
Source: JMIR Hum Factors. 2025 Mar 14;12:e65228. doi: 10.2196/65228 (PMC11953604; doi:10.2196/65228)
Supplement: Multimedia Appendix 2 [file humanfactors_v12i1e65228_app2.docx]

**Plain Language Information Statement/pilot study**

Institute of Health and Wellbeing

| PROJECT TITLE: | Sleep Best-i: An online cognitive-behavioural intervention for the treatment of insomnia and nightmares in bushfire survivors |
| --- | --- |
| PRINCIPAL RESEARCHER: | Professor Gerard Kennedy |
| OTHER/STUDENT RESEARCHERS: | Fadia Isaac, Dr Samia R Toukhsati, Professor Britt Klein, Dr Mirella Di Benedetto |

**Dear participant,**

You are invited to participate in this research project. Please read this sheet carefully so you are confident that you understand the contents before deciding whether to participate. If you would like further information about this project, please contact the principal researcher via email: [g.kennedy@federation.edu.au](mailto:g.kennedy@federation.edu.au).

***What is the purpose of this project?***

This research project aims to assess the efficacy of a digital mental health intervention program (Sleep Best-i) for the treatment of insomnia and nightmares in wildfire/bushfire survivors.

***What does this study involve?***

To be eligible to take part in this study, you need to be 18+ years of age, have been exposed to wildfires/bushfires in the past, have sufficient knowledge of the English language, have access to a computer, iPad or a smart phone and have access to the internet.

Potential participants will answer online questions that are likely to take 5-10 minutes in duration. The questions include demographic information, questions about wildfires/bushfires, current medications, mental health history, questions that assess sleeping difficulties, trauma symptoms and questions about nightmares. Eligible participants will have to sign a consent form. Those signing the consent form will complete other online questions that will take 5 minutes to complete. The online questions assess symptoms of depression, anxiety and sleep quality. Participants will also be offered the opportunity to attend a clinical interview with a clinical psychologist to establish diagnosis of sleep disorders ***should they wish***. The clinical interview, 15 minutes in duration, will ask questions about sleep difficulties to establish diagnosis of sleep disorders, trauma symptoms, and questions about medications. The clinical psychologist running the clinical interviews, has 13 years of experience and has received training in diagnosing and treating sleep and trauma symptoms. Eligible participants will be assigned to either an intervention group or a waitlist group. The intervention group will receive treatment immediately and the waitlist group will start receiving treatment after 4 weeks.

The intervention group will fill out a sleep diary, 5 minutes to complete, once a week during the treatment. The sleep diary includes questions about time going to bed, hours spent in bed, and quality of sleep. A Fitbit Inspire 2 will be posted to participants (Australian participants only) to measure sleep objectively. Participants will be asked to wear their Fitbit throughout the study. Participants will be given 6 modules of Sleep Best-i (each 17 minutes in duration) to implement weekly for 4 weeks as part of their treatment. Participants will also be given one relaxation module to use as part of their online intervention. Those who complete the treatment and provide data, will keep the Fitbit Inspire 2 (Australian participants only) as a thank you for their contribution in the study. All participants who complete the study will receive a $100 Coles e-voucher (Australian participants), and a $100 Amazon voucher (participants from the USA and Canada). Automated emails will be sent at week 2, week 3 and week 4 to address any concerns or answer questions, and get feedback on the intervention (15 minutes to complete). At the end of week 4 and week 12, participants will complete self-administered questionnaires (20 minutes in duration). The waitlist group will start the digital mental health intervention/Sleep Best-i following a four-week wait period. Potential participants will get access to all materials associated with this study via a platform called HealthZone at Federation University Australia.

This study has been approved by the Federation University Human Research Ethics Committee (approval number: 2022-153).

***Do I need to sign a consent form for this study?***

Yes, a written consent is required for participation in the study. However, participation in this study is voluntary and there is no obligation to be involved, and no explanation is needed for refusal to participate.

***What will happen if I decide to withdraw my consent?***

Participants are free to withdraw their consent at any time during the recruitment phase, during the treatment phase or when the study has concluded. There will be no consequences to participants if they do not wish to answer any questions that they don’t feel comfortable in responding to. Please also note that withdrawing a consent after the data has been combined, grouped and processed, it will not be possible to withdraw participants’ non-identifiable data, however, participants can still withdraw their consent.

***What are the possible risks or disadvantages associated with participation?***

Participating in this study may trigger pervious trauma or it may lead to reliving of events related to the experience of wildfires/bushfires. Furthermore, the materials presented in the modules may also cause distress or discomfort to some people. If you feel distressed while taking part in this project, participants in Australia are encouraged to contact their contact their general practitioner, mental health provider, or contact Beyondblue on (1300 22 4636). Email and chat service is also available <https://beyondblue.org.au/get-immediate-support>. They can also speak with a registered nurse by calling the healthdirect hotline on (1800 022 222) from anywhere in Australia. The hotline is open 24 hours, 7 days a week. If you have thoughts of suicide, please contact Lifeline on (13 11 14) or Suicide Call Back Service on (1300 659 467).

Participants in Canada are encouraged to contact their general practitioner, mental health provider, or Canada Suicide Prevention Service (CSPS) on **1-833-456-4566, or send a text to 45645 available from 4:00 pm to midnight, should you become distressed. You can also contact Hope for Wellness Helpline on 1-855-242-3310 or connect to online Hope for Wellness chat through hopeforwellness.ca.**

**Participants in the USA are advised to contact their** general practitioner, mental health provider, or contact Suicide & Crisis Lifeline on 988, or chat live by texting HELLO to 741741 should you become distressed as a result of participating in this study.

If you have any questions about this project, please contact Professor Gerard Kennedy on (61) 3 5327 6651 during business hours or via email at: g.kennedy@federation.edu.au. Prof Kennedy will discuss your questions with you confidentially and suggest appropriate follow-up, if necessary.

***What are the possible benefits associated with participation?***

The cognitive and behavioural components used in Sleep Best-i are well researched and highly effective in reducing the symptoms of insomnia, nightmares, and trauma. Participants in Australia will keep the Fitbit Inspire 2, and receive a $100 Coles e-voucher upon completion of the study. Participants in the USA and Canada will receive a $100 Amazon voucher upon completion of the study.

***Methods of data collection, storage and dissemination?***

Even though, personal information such as name, age, email address and a mobile number will be collected, participants will not be personally identified in any publications arising from the study. Collected data will be retained in secure files on a computer system protected by a password known to the researchers of this project only. Data will be stored separately from any listing that includes personal or other identifying information.

The site we are using for collecting data is Qualtrics. When the study is open and running, data will be stored within Qualtrics. After the closing date of the study, data will be exported from Qualtrics and deleted from the Qualtrics system. Data will then be retained in secure files on a computer system protected by a password known to the researchers of this project only. All data will be kept for 5 years before being destroyed permanently.

Group data will be used by the researchers of this project to assess the effectiveness of the intervention for the treatment of insomnia and nightmares. Findings from the study will form a part of a researcher’s PhD degree, and therefore findings will be presented in a thesis, may be published in scientific journals, as well as presented in conferences held nationally and internationally. No identifying personal information will be revealed in any publications arising from this study.

Please note that in the event of a small number of participants, there is a slight risk that some data may be identifiable, which could have anonymity implications. In addition, the confidentiality of information provided is subject to legal limitations (e.g., subpoena, freedom of information claim, or mandatory reporting in some professions).

***Can I review results, data, or publication arising from this research?***

As a participant of this project, you have the opportunity to preview outcome results and any publications arising from this project. Please contact Professor Gerard Kennedy on [g.kennedy@federation.edu.au](mailto:g.kennedy@federation.edu.au) should you wish to receive a summary of the project and outcomes or publications related to this study.

***Can I amend previously provided data/answers to certain questions?***

Participants can amend previously provided data to questions when the study is still open and running. However, when the study’s data have been combined and grouped, it will not be possible to amend answers because it will not be possible to change non-identifiable data.

***Whom should I contact if I have any questions?***

Questions about this study should be directed to Professor Gerard Kennedy on (+61) 3 5327 6651 during business hours or via email at: [g.kennedy@federation.edu.au](mailto:g.kennedy@federation.edu.au).

***What should I do next if I want to take part in this study?***

To take part in this study, please answer the online questions to follow this plain language statement. Once your eligibility/or not for this study is established by the researchers, you will receive an email to confirm if you can participate or not in the study.

| If you have any questions, or you would like further information regarding the project titled (Sleep Best-i: An online cognitive-behavioural intervention for the treatment of insomnia and nightmares in bushfire survivors), please contact Professor Gerard Kennedy on (+61) 3 5327 6651 during business hours or via email at: [g.kennedy@federation.edu.au](mailto:g.kennedy@federation.edu.au). Institute of Health and Wellbeing, Federation University, Ballarat, Victoria, Australia*.* |
| --- |
| Should you (i.e., the participant) have any concerns about the ethical conduct of this research project, please contact the Federation University Coordinator Research Ethics, Research Services, Federation University Australia,  P O Box 663 Mt Helen Vic 3353  Telephone: (+61) 3 5327 9765  Email: [research.ethics@federation.edu.au](mailto:research.ethics@federation.edu.au)  CRICOS Provider Number 00103D |
