# Supplementary material for: Digital Cognitive Behavioral Therapy–Based Treatment for Insomnia, Nightmares, and Posttraumatic Stress Disorder Symptoms in Survivors of Wildfires: Pilot Randomized Feasibility Trial
Source: JMIR Hum Factors. 2025 Mar 14;12:e65228. doi: 10.2196/65228 (PMC11953604; doi:10.2196/65228)
Supplement: Multimedia Appendix 3 [file humanfactors_v12i1e65228_app3.pdf]

Consent Form

Human Research Ethics Committee

http://www.federation.edu.au/

**PROJECT TITLE:**<br><br>**ETHICS APPROVAL #**	Sleep Best-i: An online cognitive-behavioural intervention for the treatment of insomnia and nightmares in bushfire survivors<br><br>2022-153
**RESEARCHERS:**	Professor Gerard Kennedy, Fadia Isaac (PhD Candidate), Dr Samia R Toukhsati, Professor Britt Klein, Dr Mirella Di Benedetto

Consent – Please complete the following information:

I ____ of

____+

Phone number____________________

hereby consent to participate as a subject in the above research study.

The research program in which I am being asked to participate has been explained fully to me, verbally and in writing, and any matters on which I have sought information have been answered to my satisfaction.

I understand that: all information I provide (including questionnaires) will be treated with the strictest confidence and data will be stored separately from any listing that includes my name and address.

- Aggregated results will be used for research purposes and may be reported in scientific and academic journals.
- I am aware that in participating in this research, my collected data will be stored securely for 5 years before being destroyed permanently.
- I am aware that in the event of a small number of participants, there is a slight risk that some data may be identifiable, which could have anonymity implications.
- The confidentiality of information provided is subject to legal limitations (e.g., subpoena, freedom of information claim, or mandatory reporting in some professions).
- I will attend a clinical interview with a clinical psychologist (15 minutes in duration) to establish diagnosis of sleep disorders ***if I wish***.
- I will fill out a sleep diary once a week for four weeks for the intervention group, and eight weeks for the wait list group.

- I will fill out a set of online self-administered questionnaires at the start of the treatment (5 minutes in duration), at week 4 of treatment and week 12 after treatment (20 minutes in duration).
- I will receive 6 modules of an online self-paced intervention (Sleep Best-i) to administer over 4 weeks (each module is 17 minutes in duration) as a treatment for insomnia and nightmare disorder.
- In week 1 and week 4 of my treatment, I will receive two modules instead of one.
- I will receive automated emails at the end of week 2, week 3 and week 4 of the treatment to get my feedback on the modules.
- For participants in Australia ONLY: I will be posted a Fitbit Inspire 2 to wear for the entire duration of the study.
- I am aware that the researchers will provide instructions about the sleep diary, and how to use the Fitbit Inspire 2 at the start of the treatment.
- To participants in Australia ONLY:I am aware that I will return the` Fitbit Inspire 2 to the researchers if I decide to discontinue the study.
- For Australians' participants ONLY: I am aware that I get to keep the Fitbit Inspire 2 if I complete the treatment.
- I am aware that I will receive a $100 Coles or Amazon vouchers upon me completing the study
- I will be given access to a platform called HealthZone at Federation University Australia to access all materials and modules related to this study.
- I am free to withdraw my consent at any time during the study in which event my participation in the research study will immediately cease and information/data obtained from it will not be used.
- I understand the exception to this is if I withdraw after information has been aggregated - it is unable to be individually identified - so from this point it is not possible to withdraw my information/data, although I may still withdraw my consent to participate.

SIGNATURE:___________________________________ **DATE:** ____________________.
